# Prevalence Trends of Foodborne Pathogens *Bacillus cereus*, Non-STEC *Escherichia coli* and *Staphylococcus aureus* in Ready-to-Eat Foods Sourced from Restaurants, Cafés, Catering and Takeaway Food Premises

**DOI:** 10.3390/ijerph21111426

**Published:** 2024-10-27

**Authors:** Nicole Foxcroft, Edmore Masaka, Jacques Oosthuizen

**Affiliations:** Occupational and Environmental Health, Medical and Health Sciences, Edith Cowan University Joondalup, Perth 6017, Australia; e.masaka@ecu.edu.au (E.M.); j.oosthuizen@ecu.edu.au (J.O.)

**Keywords:** *Bacillus cereus*, non-STEC *Escherichia coli*, *Staphylococcus aureus*, ready-to-eat foods, restaurants, cafes, catering, prevalence, linear trends

## Abstract

Foodborne pathogens of *Bacillus cereus* (*B. cereus*), non-STEC *Escherichia coli* (non-STEC *E. coli*) and *Staphylococcus aureus* (*S. aureus*) are currently non-notifiable in Australia unless attributed to a food poisoning outbreak. Due to the lack of data around individual cases and isolations in foods, any changes in prevalence may go undetected. The aim of this study was to determine any changes in the prevalence of *B. cereus*, non-STEC *E. coli* and *S. aureus* in ready-to-eat (RTE) foods sampled from Western Australian restaurants, cafés, catering facilities and takeaway food premises from July 2009 to June 2022. A total of 21,822 microbiological test results from 7329 food samples analysed over this 13-year period were reviewed and analysed. Linear trend graphs derived from the annual prevalence and binary logistic regression models were used to analyse the sample results, which indicated an increase in prevalence for *B. cereus.* In contrast, a decrease in prevalence for both *S. aureus* and non-STEC *E. coli* was determined. Additionally, there were changes in prevalence for the three bacteria in specific months, seasons, specific RTE foods and food premises types. Further research is needed to gain a better understanding of the potential drivers behind these changes in prevalence, including the potential impacts of climate change, COVID-19, legislation and guidelines targeting specific RTE foods, and the difficulty of differentiating *B*. *cereus* from *B. thuringeniesis* using standard testing methods.

## 1. Introduction

Foodborne illnesses represent a significant global public health challenge, posing substantial risks to consumer health and imposing economic burdens on healthcare systems worldwide [1]. Millions of foodborne diseases are reported yearly, attributed to a diverse array of microbial pathogens that contaminate various types of ready-to-eat (RTE) foods [1,2,3]. Among these pathogens, *Bacillus cereus* (*B. cereus*), *Escherichia coli* (*E. coli*) and *Staphylococcus aureus* (*S. aureus*) have continued in importance due to their association with foodborne outbreaks and their potential to cause severe illness [1]. Despite challenges in data collection for *B. cereus*, non-Shiga toxin-producing *Escherichia coli* (non-STEC *E. coli*) and *S. aureus*, specifically around disease reporting, the WHO highlighted their substantial impact based on data from European and other subregion A countries, underscoring their significance in food safety [1]. The Foodborne Disease Outbreak Surveillance System (FDOSS) has been pivotal in tracking foodborne illness outbreaks in the United States. Data from CDC reports between 2011 and 2017 revealed fluctuations in outbreaks associated with *B. cereus*, *S. aureus*, and non-STEC *E. coli*, providing insights into their epidemiological patterns and impact on public health [4,5,6,7,8,9,10]. Similarly, in Europe, the European Food Safety Authority (EFSA) has been instrumental in monitoring foodborne diseases within the European Union (EU). Recent reports indicate varying outbreaks linked to *B. cereus toxins*, *S. aureus*, and *E. coli* across member states, highlighting regional disparities and the challenges in ensuring uniform food safety standards [2].

Australian food safety regulators have completed snapshot surveys by sampling from different food products [11,12,13,14,15,16]. In addition, they have data about foodborne disease investigations and outbreaks. However, there is little published information available for sampling programs monitoring specific food premises and similar RTE foods over long periods of time that monitor *B. cereus*, non-STEC *E. coli* and *S. aureus*. These three bacteria are important in assessing the microbiological safety and condition of RTE foods, and results above satisfactory limits can provide an indication of the handling and hygiene of the product or potential issues in source ingredients or chain of supply. In Western Australia, these three bacteria are non-notifiable to the state and/or local government if isolated in food samples or via infection (under the Notifiable Communicable Disease requirements) unless the strain is defined as a Shiga toxin-producing *E. coli* (STEC) [17]. Ongoing data from industry microbiological monitoring programs for RTE foods are rarely shared between industry and food safety regulators, especially results above satisfactory limits that do not trigger a notifiable event. The long-term tracking and trending of *B. cereus*, non-STEC *E. coli* and *S. aureus* in RTE foods can provide information on any significant changes in prevalence, including specific food types. Such information could provide an early indicator of changing factors in the environment/food chain/food handling and have the potential to impact food safety practices and standards.

Internationally, governments rely on expert advice and guidance from organisations such as the Codex Alimentarius Commission to establish microbiological criteria for RTE foods [18]. The Codex adopts a risk-based approach that aligns microbiological limits with public health outcomes, advocating for the use of quantitative microbial risk assessment (QMRA) and hazard analysis critical control point (HACCP) principles to enhance food safety practices [19]. The COVID-19 pandemic, declared a Public Health Emergency of International Concern by the WHO in early 2020, has further underscored the intersection of public health emergencies and food safety [20,21]. Stringent measures such as lockdowns and enhanced hygiene practices have been implemented globally to curb the spread of the virus, impacting food supply chains, producers and consumer behaviours. The pandemic’s influence on foodborne disease outbreaks and microbial prevalence in RTE foods remains an area of ongoing research and concern [20,22]. Furthermore, the effect of climate change and extreme weather events on foodborne pathogen adaptability, growth and distribution is an area of complexity that has many factors, and more research in this area has been recommended [23].

The aim of this study was to gain an understanding of any changes in the prevalence of *B. cereus*, *E. coli* (non-STEC/generic) and *S. aureus* in RTE foods over a period of 13 years, and the potential public health implications, through the investigation of four research questions:

1. Do the target microbes exhibit annual variations in prevalence that exceed applicable satisfactory standards as defined in the Food Standards Australia and New Zealand (FSANZ) Compendium of Microbiological Criteria and the Alberta Health Services Microbial Guidelines for Ready-to-Eat Foods?
2. Do the target microbes exhibit monthly or seasonal prevalence trends in particular months and seasons?
3. Do the target microbes exhibit prevalence trends within individual RTE food categories?
4. Do the target microbes exhibit prevalence trends in individual food premises types?

## 2. Materials and Methods

A retrospective study of an industry database of microbiological test results from RTE food samples collected from restaurants, cafés and takeaway food premises in Western Australia as part of food sampling verification and validation programs from July 2009 to June 2022 was completed. This study involved the review and analysis of 21,822 microbiological test results obtained by industry from 7329 food samples of RTE foods. Permission to access the industry database was granted for the purposes of this research project, which was evaluated under the Edith Cowan University ethics approval system and determined not to require ethics approval (REMS NO: 2023-04684-FOXCROFT) since it did not involve research on human and animal subjects. The data was de-identified for privacy and ethical reasons. The definition of RTE foods for the purpose of the study is “for which it is reasonably foreseeable the food will be eaten without further processing that significantly minimizes biological hazards” [24].

The microbiological test results analysed in this study were produced by the National Association for Testing Authorities (NATA) Accredited Laboratories after testing samples collected from premises participating in the sampling program. All the samples analysed were collected by trained personnel in accordance with applicable Australian standards. The following validated culture methods in Table 1 were used for the microbiological analysis of the samples.

The microbiological test results were compiled into the database by one person to ensure consistency, accuracy and the reliability of the results. The database was extensively reviewed by the primary researcher prior to analysis to ensure the following:

- Results were entered correctly;
- Exclusion of results from any non-ready-to-eat foods, such as raw items or products being tested mid-process;
- Exclusion of food samples that did not include testing for *B. cereus* and *S. aureus* or *E. coli.*

The final database consisted of 7329 food samples with 7319 *E. coli*, 7267 *S. aureus* and 7227 *B. cereus* individual tests.

The study included 34 food premises, with each being coded with a specific food outlet number and a premises type. This premises type was determined on the style of service provided and included a la carte, buffet, catering, café, fast food takeaway or supply. The data were coded to match the operational financial years, i.e., year 1 was 2009–2010, year 2 was 2010–2011 etc., adding up to a total of 13 years of study. A binary number of either 0 for results that were within a satisfactory range or 1, for results which were above the satisfactory limit was allocated as defined in Table 2. The determination of acceptable microbiological levels for foodborne pathogens in RTE foods has been more specifically defined in countries such as Australia, Canada, the United Kingdom and Hong Kong. There is consistency for *E. coli* STEC limits and what is concluded to be an unsatisfactory indicator *E. coli* level. However, there are variances between the different standards for what is an acceptable satisfactory level for *E. coli*. The Food Standards Australia and New Zealand (FSANZ) Compendium of Microbiological Criteria [24] for *E. coli* has a satisfactory limit of <3 cfu/g; however, the limit of testing at the time of the study was 10 cfu/g, and therefore this is the level used for this study. The Alberta Health Services Microbial Guidelines for Ready-to-Eat Foods have set a <10 cfu/g satisfactory level for *E. coli* and have similar limits for marginal and unsatisfactory results.

The majority of food samples consisted of numerous ingredients and were mixed items. The samples were categorised based on the type of cuisine and main/characterising ingredients(s). This approach was applied consistently to similar types of samples to reduce variability. The categories are defined in Table 3.

Due to some variety in the food premises surveyed over the 13 years, where some premises closed or rebranded and additional businesses were included, the food premises involved in the study were categorised into specific types of outlets based on the style of operation as follows:

1. A la carte-style operations where a range of items on menus was offered and involved significant food handling;
2. Buffet-style, where meals were offered as a self-service offering;
3. Catering, where meals were offered in a large group situation;
4. Café operations where a simple menu was offered and involved only limited food handling (storage and warming of items);
5. Fast food operations where a range of items were offered, and no table service was supplied;
6. Supply chain tests of products being sourced for all types of premises in the study.

The data set was collated in Excel version 2021 and used to analyse prevalence and produce trend graphs. SPSS Statistics Version 28.0.1.0 was used for the binary logistic regression models and Pearson correlation analysis. However, this statistical package was limited in its ability to produce a post hoc analysis to identify particular differences between groups. To overcome this limitation, the same data and logistic regression models were analysed in R (version 4.3.1, run in RStudio 2023.12.1 Build 402) to determine the differences between groups, with Tukey’s method being used for adjusting for multiple comparisons. Table 4 describes the methods used to examine each of the research questions.

To examine the research questions, the prevalence in each year, month, season, food category and food premises type for *E. coli*, *S. aureus* and *B. cereus* was calculated by the total number of results above the satisfactory limit as the numerator and the total number of tests completed for each of these bacteria as the denominator. Confidence intervals at 95% were calculated to provide insights into the prevalence results. The results were graphed for visual comparison, and linear trend lines plotted. Secondly, binary logistic regression analysis models were used to test for a linear change in prevalence of non-STEC *E. coli*, *S. aureus* and *B. cereus* over the 13 years. The food category and outlet type were included as covariates. Separate models were run for the prevalence of each bacteria. If a *p*-value of <0.05 was obtained, then the null hypothesis of no linear change in prevalence over time was rejected. The Odds Ratio for the per-year change in prevalence was obtained by taking the exponent of β (exp(β)), the coefficient of the linear relationship between year and prevalence estimated from the model. If the Odds Ratio was greater than 1, this indicated increasing per-year prevalence, while an Odds Ratio less than 1 indicated declining prevalence over time. To test for variation among years, the binary logistic regression model was repeated with year as a categorical (rather than a continuous) variable. Where there was significant variation among years (*p* < 0.05), a Tukey post hoc test was used to determine which years differed in prevalence of the bacteria. Pearson Correlation tests were used to explore the strength and direction of linear relationships between non-STEC *E. coli*, *S. aureus* and *B. cereus* prevalence over the 13 years of the study. If a *p*-value of <0.05 was obtained, then the null hypothesis of no correlation was rejected. Thirdly, binary logistic regression analysis models were used to test for a linear change in the prevalence of non-STEC *E. coli*, *S. aureus* and *B. cereus* (separate models for each bacteria) over the 13 study years, within each month, season, RTE food category and food premises type. If a *p*-value of <0.05 was obtained, then the null hypothesis of no change in prevalence over time was rejected. An Odds Ratio greater than 1 indicated an increasing per-year prevalence of the bacteria within that month, season, food category or premises type, while an Odds Ratio less than 1 indicated a declining prevalence.

## 3. Results

A total of 95.12% (n = 7329) (95% CI: 92.94% to 97.30%) of the RTE foods sampled were found to be within satisfactory limits, with 4.88% (n = 358) (95% CI: 4.77% to 4.99%) of these foods being above the satisfactory limit. Four of these samples had two bacteria isolated above the satisfactory limit in the same sample. A total of 21 822 individual tests were completed for the 7329 food samples over the 13 years, and in summary the results were as follows:

- A total of 7319 were tested for *E. coli*, with 50 identified as being above the satisfactory limit, equalling a prevalence rate of 0.68% (95% CI: 0.67% to 0.70%);
- A total of 7276 were tested for *S. aureus*, with 72 identified as being above the satisfactory limit, equalling a prevalence rate of 0.99% (95% CI: 0.97% to 1.01%);
- A total of 7227 were tested for *B. cereus* with 240 identified as being above the satisfactory limit, equalling a prevalence rate of 3.32% (95% CI: 3.24% to 3.40%);
- From the total of 21,822 combined tests for all three bacteria, 362 were identified as being above the satisfactory limit, equalling a prevalence rate of 1.66% (95% CI: 1.64% to 1.68%).

The prevalence of *S. aureus* occurring in RTE foods above the satisfactory limit over the 13 years of the study was 1.5 times that of *E. coli*. Additionally, the prevalence of *B. cereus* was 5.0 times higher than that of *E. coli*.

**Figure 1:**
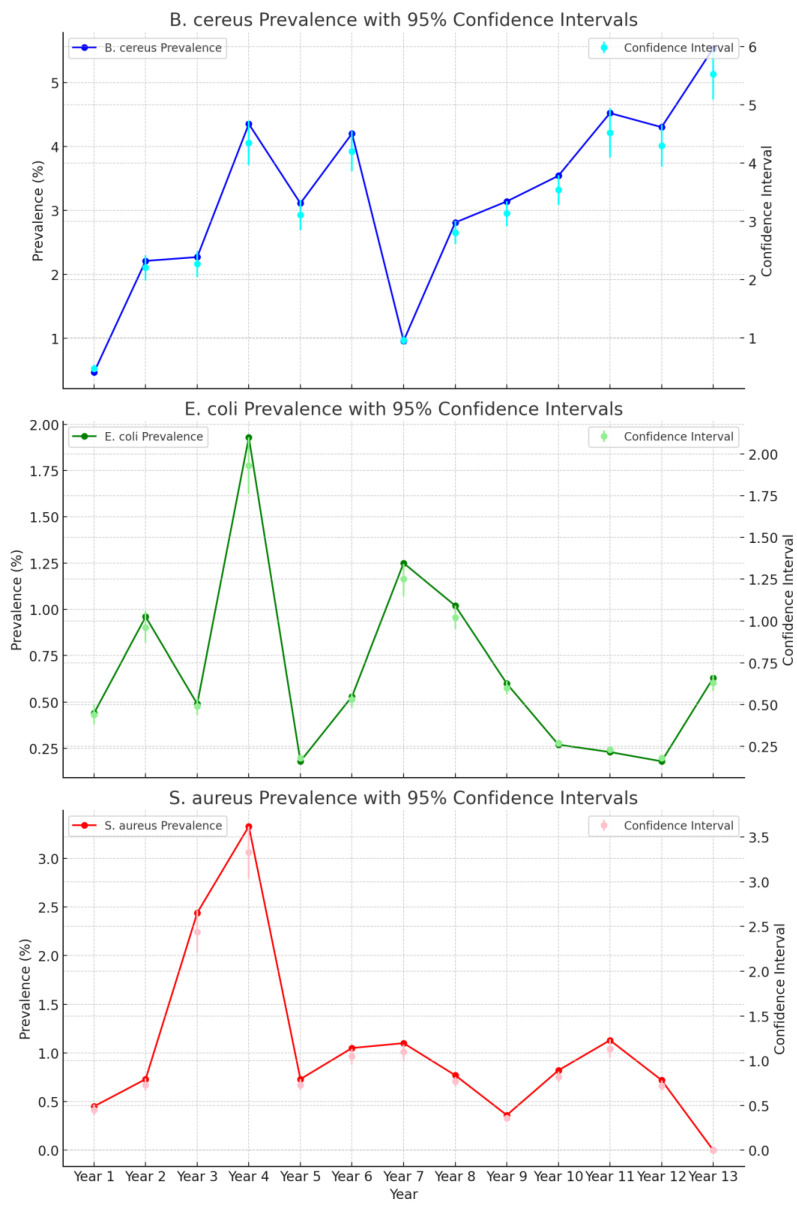
Annual prevalence trend of *B. cereus*, non-STEC *E. coli* and *S. aureus* and above satisfactory limits in ready-to-eat foods from July 2009 to June 2022.

There was an increase in *B. cereus* and a decrease in *S. aureus* and *E. coli* in *RTE* over the 13 years, as indicated in Figure 1. The prevalence rate of *E. coli* above the satisfactory limit was highest in the year of 2012–13 at 1.93% (n = 518) (95% CI: 1.76% to 2.10%), followed by 2015–16, where the prevalence rate was 1.25% (n = 642) (95% CI: 1.15% to 1.34%). The prevalence rate of *S. aureus* above the satisfactory limit was highest in the year 2012–13 with 3.33% (n = 510) (95% CI: 3.04% to 3.62%), followed by 2011–12 with 2.44% (n = 409) (95% CI: 2.21% to 2.68%). The prevalence rate of *B. cereus* above the satisfactory limit was highest in the year 2021–22 5.53% (n = 615) (95% CI: 5.09% to 5.97%), with a peak in 2012–13 4.35% (n = 506) (95% CI: 3.97% to 4.73%). The annual prevalence calculations of all three bacteria including confidence intervals are shown in Supplementary Table S1. Overall, the year 2012–13 had the highest combined prevalence rate for all three bacteria, with results above the satisfactory limit of 3.19% n = 1534 (95% CI: 3.03% to 3.35%).

The combined annual prevalence trend of *B. cereus*, *non-STEC E. coli* and *S. aureus* above satisfactory limits in ready-to-eat foods from July 2009 to June 2022 is shown in Supplementary Figure S1.

The binary logistic regression models testing for a linear association of the year (as a continuous variable) with the prevalence of *B. cereus*, *E. coli* and *S. aureus* supports what can be observed in Figure 1. The models indicated a significant positive linear change in *B. cereus* prevalence over time (OR. 1.083; 95% CI, 1.040–1.127, *p* = <0.001) with an average per-year increase of 8.3%, while there was a significant negative linear change in non-STEC *E. coli* (OR. 0.910; 95% CI, 0.836–0.991, *p* = 0.029) and *S. aureus* (OR. 0.872; 95% CI, 0.810–0.939, *p* = <0.001), with an average per-year decrease of 9.0% and 12.8%, respectively.

In addition, there was significant variation among food categories and outlet types for the prevalence of *B. cereus* (food category: Wald statistic = 43.665, df = 19, *p* = 0.001; outlet type: Wald statistic 13.208, df = 5, *p* = 0.022) and *S. aureus* (food category: Wald statistic = 47.842, df = 19, *p* = <0.001; outlet type: Wald statistic 21.921, df = 5, *p* = <0.001), but not for *E. coli* (food category: Wald statistic 10.669, df = 19, *p* = 0.934; outlet type: Wald statistic 3.537, df = 5, *p* = 0.618). Table 5 highlights the significant differences from the post hoc analysis for *B. cereus* and *S. aureus* within the food categories and outlet types. However as there was no significant variation for *E. coli* from the same analysis, there are no inclusions in the table. All three bacteria showed significant variations within the individual years: *B. cereus* (Wald statistic = 32.481 df = 12, *p* = 0.001), non-STEC *E. coli* (Wald statistic = 23.129, df = 12, *p* = 0.027) and *S. aureus* (Wald statistic = 30.141, df = 12, *p* = 0.003). The post hoc analysis indicated a significant difference between the 2021–22 and 2011–12 periods, with the isolation of *B. cereus* above the satisfactory limits being higher in the former period. In contrast, a significant decrease in the isolation of *B. cereus* above the satisfactory limits was determined in the year 2015–16 compared to the three years from 2012–13 to 2014–15, with a similar trend being seen with *S. aureus* between the 2021–22 and 2013–14 periods. The large standard error relating to *S. aureus* outputs on the post hoc may be attributed to the non-detection of this bacteria in the years 2021–22 and within the fried food category.

A significant positive correlation in the prevalence of *E. coli* and *S. aureus* in RTE was observed over the 13-year sampling period (r = 0.2, *p* = 0.022, 95% CI 0.004–0.050); however, the Pearson correlation value was less than 0.3.

Table 6 shows the monthly and seasonal variations in the occurrence of *B. cereus*, non-STEC *E. coli* and *S. aureus* in the RTE investigated in this study. The occurrence of non-STEC *E. coli* did not vary significantly across the months; however, there was a significant decrease observed during the Australian summer period. The prevalence of *S. aureus* declined over the years of the study in the months of May (OR 0.694; 95% CI, 0.508–0.948, *p* = 0.022) and October (OR 0.700; 95% CI, 0.531–0.922, *p* = 0.011); however, the prevalence of *B. cereus* increased significantly with the years during the months of January (OR 1.157; 95% CI, 1.033–1.295, *p* = 0.012), September, (OR 1.192; 95% CI, 1.034–1.375, *p* = 0.016) and November (OR 1.196; 95% CI, 1.026–1.395, *p* = 0.022). The occurrence of *B. cereus* in spring each year increased (OR 1.138; 95% CI, 1.057–1.226, *p* = <0.001), while *S. aureus* decreased (OR 0.789, 95% CI 0.680–0.917, *p* = 0.002). The annual prevalence trends for *B. cereus*, *E. coli* and *S. aureus* in the Australian spring is shown in Supplementary Figure S2.

The prevalence calculations with confidence intervals of *B. cereus*, *E. coli* and *S. aureus* for each month and season is shown in Supplementary Tables S2 and S3.

Table 7 shows the prevalence of *B. cereus* in the individual food categories across the four Australian seasons. Mixed salads/cold RTE food samples had high prevalence rates for *B. cereus* during the autumn 8.97% (n = 123) (95% CI: 7.57% to 10.38%) and spring 6.85% (n = 146) (95% CI: 5.74% to 7.96%) seasons. The prevalence of *B. cereus* was highest in cheeses/smallgoods/platters during the winter season (7.04% (n = 71) (95% CI: 5.40% to 8.68%)) and second highest in the spring season (6.9% (n = 58) (95% CI: 5.12% to 8.67%)); however, the highest prevalence of 33% (n = 9) (95% CI: 11.56% to 55.11%) for spices during the same season can be disregarded due to the small sample and large confidence interval size. The prevalence of *B. cereus* increased significantly over the years within the food categories of mixed salads/cold RTE (OR 1.114; 95% CI, 1.009–1.231, *p* = 0.033) and cheeses/smallgoods/platters (OR 1.259; 95% CI, 1.016–1.559, *p* = 0.035). While significant linear changes in *B. cereus* prevalence over the years of the study (July 2009 to June 2022) were not observed in any other food categories, the Odds Ratios for change in *B. cereus* prevalence over time in 10 further individual food categories were greater than 1, suggesting an increasing prevalence, albeit non-significant. In contrast, the occurrence of *S. aureus* decreased within the food categories of sashimi/sushi (OR 0.730; 95% CI, 0.593–0.899, *p* = 0.003) and in pastry/desserts/bread (OR 0.643, 95% CI 0.459–0.901, *p* = 0.010).

**Figure 2:**
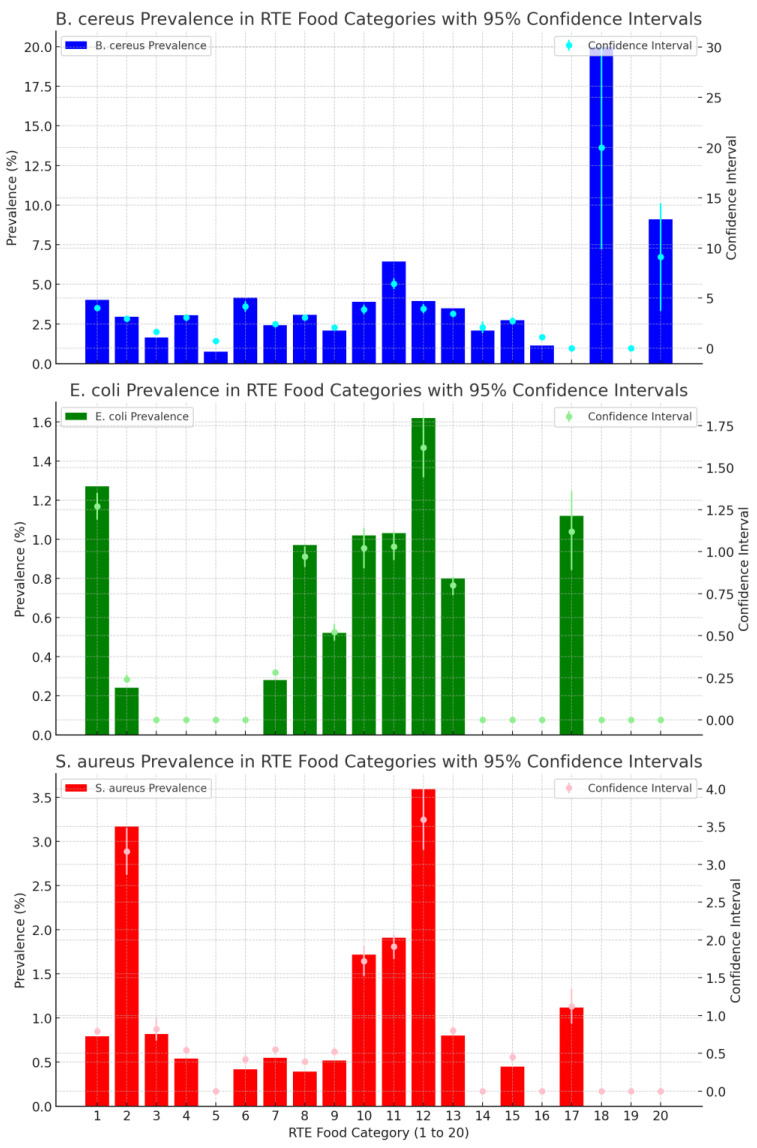
Prevalence trend of *B. cereus*, *E. coli* and *S. aureus* above satisfactory limits in RTE food categories from July 2009 to June 2022. 1.RTE Seafood, 2.Sashimi/Sushi, 3.Cooked Wet Dishes, 4.Cooked Whole Meats, 5.Soup and Stock, 6.Purees/Sauces/Dips, 7.Western Style Dishes, 8.Asian Style Dishes, 9.Rice/Noodle/Pasta, 10.Burgers/Sandwiches, 11.Mixed Salads/Cold RTE, 12. Cheese/Smallgoods, 13. Pastry/Dessert/Bread, 14. Fruit/Nuts, 15. Vegetables/Herbs, 16. Fried Foods, 17. Dairy/Eggs, 18. Spices, 19.Other, 20.Beverage.

Figure 2 shows the prevalence of *B. cereus*, *E. coli* and *S. aureus* in different food categories over a 13-year period. *B cereus* had the highest prevalence rates across the largest number of food categories in comparison to *E. coli* and *S. aureus*, with this pathogen being present in all food categories except dairy and other foods. Spices and beverages had high prevalence rates; however, they also had large confidence intervals, which relate to the small number of samples taken over the 13-year period. Mixed salads, with a prevalence rate of 6.43% (n = 575) (95% CI: 5.91% to 6.96%), had a more representative sample size. Ten (10) food categories did not have any detections of *E. coli* over the 13 years. The food categories of cheese/smallgoods (1.62% (n = 308) (95% CI: 1.44% to 1.80%)) and RTE seafood (1.27% (n = 1024) (95% CI: 1.19% to 1.35%)) had the highest percentage of *E. coli* results above satisfactory limit, with *S. aureus* also having the highest percentage of detections in cheese/smallgoods (3.59% (n = 306) (95% CI: 3.19% to 4.00%)).

The prevalence calculations with confidence intervals for each food category is shown in Supplementary Table S4.

**Figure 3:**
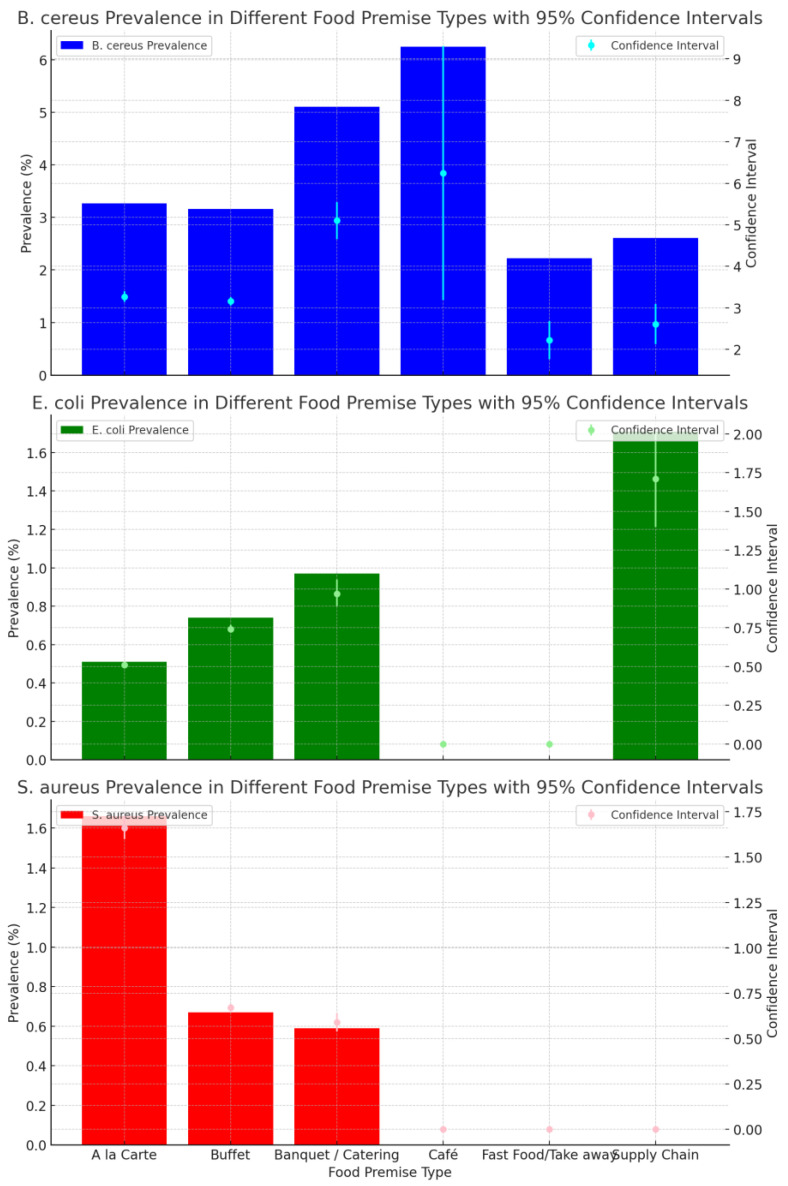
Prevalence trend of *B. cereus*, non-STEC *E. coli* and *S. aureus* above satisfactory limits in difference food premises types from July 2009 to June 2022.

Figure 3 shows the prevalence of *B. cereus*, *E. coli* and *S. aureus* for results across different food premise types. *B. cereus* was isolated in all food categories and had the highest prevalence when compared with *E. coli* and *S. aureus* in each of the different food premises types. Cafés and catering had the highest *B. cereus* prevalence, 6.25% (n = 16) (95% CI: 3.19% to 9.31%) and 5.11% (n = 509) (95% CI: 4.66% to 5.55%) respectively. *S. aureus* was isolated above the satisfactory limit in three food categories, with the highest in a la carte premises (1.66% (n = 2523) (95% CI: 1.60% to 1.73%)), whilst *E. coli* had the highest prevalence of 1.71% (n = 117) (95% CI: 0.67% to 0.70%) in the supply chain.

The detection of *B. cereus* above satisfactory limits in premises offering buffet (OR 1.096; 95% CI, 1.034–1.163, *p* = 0.002) and catering services (OR. 1.113; 95% CI, 1.004–1.233, *p* = 0.042) increased with the years (Table 8). In contrast, the detection of *E. coli* and *S. aureus* in buffet-style premises showed a decrease over the years ((OR 0.895; 95% CI, 0.802–0.998, *p* = 0.046) and (OR 0.871; 95% CI, 0.775–0.978, *p* = 0.020), respectively). Additionally, *S. aureus* also showed a decrease over the years in the a la carte category (OR 0.893; 95% CI, 0.814–0.979, *p* = 0.016).

## 4. Discussion

The prevalence of *B. cereus*, non-STEC *E. coli* and *S. aureus* in ready-to-eat (RTE) foods handled in Western Australian restaurants, cafés, catering and takeaway food premises had not previously been determined. Understanding the long-term trends of these foodborne pathogens in RTE foods is important to improve food safety and provide an early indicator of changing factors in the environment/food chain/food handling and has the potential to impact food safety practices and standards.

The high percentage of RTE samples that fell within satisfactory limits in this study reflects the high standards maintained by the food premises involved in the study and their effective food safety plans, inclusive of microbiological sampling activities. The rare occurrence of isolations above the satisfactory limit, when considered in relation to the observed linear trends, may signal a potential change in underlying conditions where there is an emerging risk or there are external influences (climate) or effective intervention measures, and warrants further investigation. Natural variability and cyclical patterns can be associated with rare events [27], hence the use of a binary logistic regression model to reduce the chance element with general prevalence calculations.

The occurrence of *E. coli* and *S. aureus* in RTE foods exhibited a weak positive correlation. This is a phenomenon that needs further investigation based on a much bigger study, considering the potential public health implications associated with the well-established food poisoning potential of these pathogens [28,29,30].

### 4.1. B. cereus

*Bacillus cereus* (*B. cereus*) forms part of industry and regulatory surveillance and monitoring testing programs in Australia, especially for cook/chill and RTE foods. *B. cereus* is an aerobic, Gram-positive, motile, spore-forming bacterium which produces toxins that can cause food poisoning [31], and it forms part of the *Bacillus cereus* sensu lato group that also includes *B. anthracis* (etiological agent of anthrax) and *B. thuringiensis* (strains used in biological control) [32]. All three are closely related, in particular *B. cereus* and *B. thuringiensis,* where gene coding for insecticidal toxins is the only established differentiation [33]. *B. cereus* is ubiquitous in nature and readily found in soil, where it adopts a saprophytic cycle of germinating, growing and sporulating [31]. *B. cereus* also grows well in the intestinal tract of insects and mammals, and it is from these environments *B. cereus* can easily spread to foods [34]. The development of spores occurs relatively soon after the cessation of growth, and germination can occur within hours of suitable growth conditions being established [35].

*B. cereus* spores are highly resistant to heat, chemical and other environmental stressors. The *B. cereus* group can survive and multiply within a wide temperature range, including psychotrophic and mesophilic domains. *B. cereus* is widely distributed in the environment [34] and displays a large variety of adaption characteristics such as the ability to spore, with a number of strains displaying mesophilic growth at optimal temperatures of 35–40 °C [36]. Furthermore, most strains are motile, tolerate salt concentrations of 7.5% and can occur in pH ranges from 4.9 to 9.3 [37]. With the expectation of increased temperatures and extreme weather events such as drought, the possibility of the inhibition of natural soil microflora may allow for more resistant groups of bacteria such as *Bacillus*, *Staphylococcus* and *Clostridium* to survive and increase in dispersion [23]. Carlin et al. (2010) discussed *B. cereus’s* current climate selection and explored the idea of its adaptation capabilities, the conclusion being that *B. cereus* be used as the model of choice when investigating the impact of global warming on microbiological populations, specifically due to its genetic structure and adaptation to temperature differences among its groups [36].

A further capability of *B. cereus* is the production of biofilms on food contact surfaces. *B. cereus*’s persistence and ability to thrive in unique niches provides an added challenge for food safety programs. *B. cereus* has a variety of biofilms, and of interest is the hypothesis that differences in the biofilm architecture and mechanism of formation may possibly reflect an adaptation to various environments [38,39]. The public health significance of *B. cereus* has already been established globally; however, its specific survival capabilities and possible environmental adaptations may become more significant for food safety regulators and industry when considering our changing climate. By the nature of their processing, and the amount of RTE foods consumed in Australia, *B. cereus* poses a significant public health risk. An increase in its prevalence in these foods further impacts this risk by potentially increasing sources of exposure and the spread of antibiotic-resistant strains. Hypothesises around *B. cereus’s* adaptability in terms of temperature range and environments highlights its importance to public health [36].

The annual prevalence of *B. cereus* in RTE foods significantly increased throughout the study duration (OR, 1.083; 95% CI, 1.040–1.127, *p* = <0.01). A review of the literature found few comparable studies with a focus on the change in prevalence of *B. cereus*, *S. aureus* and *E. coli* over time, incorporating the range of ready-to-eat foods and types of premises involved in this study [40,41,42,43,44,45,46,47,48,49,50]. However, the comparison of this research’s findings with the other studies on this subject has limitations due to the differences in the time frames covered and the sample sizes investigated. A recent study did provide some insight involving the prevalence of *B. cereus* in foods from retail food premises between 2016 and 2020 and the relationship between the percentage of disqualified samples in 2016–2020 [46]. Of the 45,358 tests completed, the analysis did not establish any statistically significant change in prevalence over the years and a low level of prevalence in the foods tested of 0.725% (n = 45,358). However, there was a significant difference in the criteria for determining the level for *B. cereus* in the prevalence calculations. The study focused on a disqualification level of >10^5^ cfu/g derived from the Poland national guidelines. This is equal to the unacceptable standard in the FSANZ Microbiological Compendium, which is significantly different from the above satisfactory level determined for this study. Further, the largest percentage of the samples (95.1% n = 43,152) were tested in confectionary and pastry products, with small numbers of samples in other food categories, which restricts any comparisons with this study’s findings.

The observed high prevalence of *B. cereus* in several of the RTE foods in this study is consistent with the findings of another study that investigated 840 RTE foods from retail markets and supermarkets in 39 major Chinese cities conducted between 2011 and 2016 and that determined a 35% (n = 870) prevalence rate of this same pathogen and a potential high risk of foodborne disease, due to the presence of a variety of pathogenic genes and multidrug-resistant profiles [49]. While this Chinese research did not include the range of food categories covered in this study, or review changes in prevalence over time, it did highlight the risk associated with a high prevalence of *B. cereus* in some RTE food types. The similar finding in this study justifies the need to further investigate strategies to better detect and control the occurrence of *B. cereus* in a wider range of RTE food categories.

When examining the results from the twenty (20) different RTE food categories, there were twelve (12) that had an increase in the linear trend of *B. cereus* across the 13 years, all with an Odds Ratio greater than 1, and significantly, in mixed salads/cold RTE and cheese/smallgoods platters, the odds of *B. cereus* being isolated above the satisfactory limit increased by approximately 11.4% and 25.9% per year. Some studies of *B. cereus*’s prevalence in milk and cheeses have highlighted an ongoing public health risk from contamination by some specific strains of this pathogen [51,52,53]. A recent study into *B. cereus* prevalence in artisan cheese in Southwestern Mexico identified a *B. cereus* contamination rate of 29.48%, n = 78, and concerningly, the strains identified had an increased toxigenic potential [40].The trend towards more artisan-type products, including raw and unpasteurised foods, may increase the risk for *B. cereus* foodborne outbreaks. This established risk of *B. cereus* contamination in milk and cheeses gives weight to the same findings determined in this study, and requires further investigation to understand the possible sources and trends of this pathogen in this food type. This study determined an increased prevalence of *B. cereus* in salads, a food type that is becoming increasingly popular due to recommendations from health professionals on dietary and health benefits, the vegetarian/vegan movement and the increased availability and convenience of readymade salads, and it is consistent with several other studies that have highlighted the public health risk caused by this pathogen in this food type [54,55,56].

The findings of this study regarding the increasing prevalence of *B. cereus* in RTE needs to be evaluated in the context of the limited ability of current laboratory methods to detect and enumerate and distinguish between *B. cereus* and *B. thuringeniesis* (*Bt*), primarily due to their genetic similarity [57]. *B. thuringeniesis* could be considered a confounder within the results, particularly in the category of mixed salads/cold RTE, where it is used as a common biological pesticide in the form of sprays/dusts/granules and pellets. Additionally, *B. thuringeniesis* has been incorporated into certain food crops to enable them to produce this microbe themselves [58]. The technology of the NATA accredited laboratories used to analyse and produce the results investigated in this study at the time could not complete a differentiation between *B. thuringeniesis* and *B. cereus*. A report by the European Commission recommended the use of whole genome sequencing to provide the unambiguous identification of strains used as biopesticides and the detailed characterisation of outbreak strains, allowing the discrimination of *B. thuringeniesis* from *B. cereus* [59].

Furthermore, the food category of mixed salads also included proteins and grains that were subjected to cook/chill processes that may have provided the opportunity for *B. cereus* growth [60], adding to the uncertainty of the impact of *B. thuringeniesis.* Therefore, the increasing trend of *B. cereus* in RTE foods observed in this study needs to be investigated further, with genome sequencing being used as part of laboratory isolation and detection methods.

This study determined that the prevalence of *B. cereus* in RTE foods was highest in the first year of the COVID-19 pandemic compared to the previous years of the study (4.52% n = 442). The two following years did not see any significant decrease, with the last year of the study having the highest annual prevalence rate, which was statistically significant compared to the 2011–2012 year. The potential impact of the COVID-19 pandemic on the observed increase in the prevalence of *B. cereus* in RTE foods is a phenomenon that also needs further investigation. Some research in the early phases of COVID-19 in the USA indicated a significant decline in the rates of foodborne disease; however, this did not include the three bacteria that were the focus of this study, making the findings inconsistent with those of this study [61]. However, the USA study highlighted the impact COVID-19 containment measures had on agriculture and food systems, such as the effects on food security, availability, access and stability [61], and is an area for further research.

**Figure 4:**
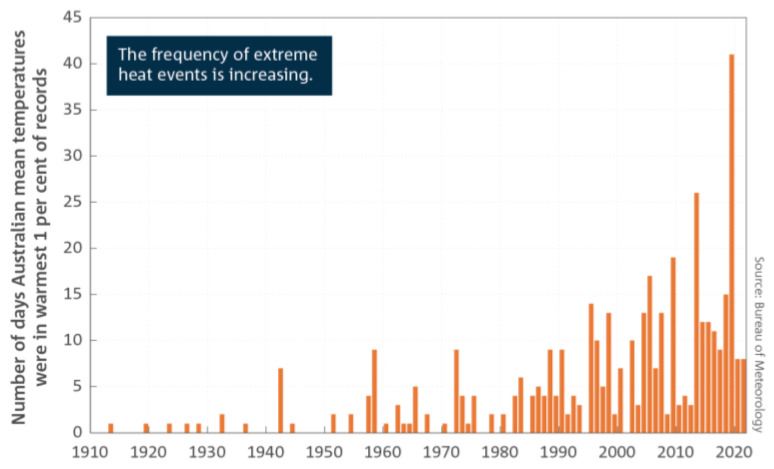
Number of days each year where the Australian area averaged daily mean temperature for each month is extreme (extremely warm days). Extremely warm days are defined as those where daily mean temperatures are the warmest 1 per cent of days for each month, calculated for the period from 1910 to 2021 [64].

The year 2015–16 had a significantly lower prevalence for *B. cereus* in RTE foods compared to the three years from 2012–2013 to 2014–2015. Furthermore, this study determined a steady increase in the prevalence of this foodborne pathogen over the years, particularly post 2016. During the same period, the planet saw one of the strongest El Nino events on record in the Pacific Ocean, which was characterized by hot and dry early months of 2016 in the north and eastern parts Australia [62,63]. This was followed by a dramatic change in weather patterns from May onwards, with heavy rain and flooding during winter and into spring [62]. Climate data in Australia up to the end of 2022 show that the subcontinent has been warming on average by 1.47 + 0.24 °C per year since national records began in 1910, and there has been an increased frequency of extreme heat events over land and sea [64], as shown in Figure 4. This global warming phenomenon has been identified as a concern for public health because it has the potential to influence the extent and natural spread of foodborne pathogens [36]. It is argued in this study that the observed increase in the prevalence of *B. cereus* may be a function of climate change; however, further research is required to understand this phenomenon due to the complexity associated with efforts to establish the effects of climate change and the survival and dispersion of foodborne pathogens [23].

The high prevalence rates for *B. cereus* in the cheese/smallgoods/platters (6.9% (n = 58)) and mixed salads/cold RTE (6.8% (n = 146)) categories were considered in relation to their impact on the Australian spring season results. However, they have a higher prevalence in other seasons, in particular winter for cheese/smallgoods/platters and in autumn for mixed salads/cold RTE. While these three categories influenced the results for spring, five other categories had prevalence rates over 4.5%, which also contributed to the high prevalence rate. These results were more indicative of *B. cereus* prevalence over many of the categories over the 13 years of the study. Additionally with the highest prevalence rates for *B. cereus* in mixed salads/cold RTE in both autumn and spring, where there is a change in weather that may facilitate growth or dispersion, is an area for further study [23,65]. Another impact on the seasonal variations observed in the prevalence of *B. cereus* in salad food types may be related to the seasonal production of leavy vegetables during autumn and spring, as well as the possible confounding effect of *B. thuringeniesis* as discussed earlier.

The observed increase in the prevalence of *B. cereus* in cafés and catering premises could be considered in the context of the contaminating potential of this foodborne pathogen in these settings, as previously determined for rice and starchy foods [50]. This previous study also identified some common risk factors from this style of operation, including incorrect temperature control and improper cleaning of food equipment. Inadequate temperature controls, specifically around heating and cooling, were also identified in an outbreak of suspected emetic and diarrhoeal syndromes of *B. cereus* foodborne poisoning in an Australian restaurant offering a multi-course dinner in 2018 [66]. The recently introduced Standard 3.2.2A—Food Safety Management Tools by FSANZ in December 2023 has addressed requirements for monitoring and recording cooking, heating and cooling temperatures, cleaning schedules and the appointment and training of Food Safety Supervisors for each food business handling unpackaged RTE foods. The impact of this new standard on the ongoing prevalence of *B. cereus* and other foodborne pathogens in RTE foods from restaurants, cafés, banqueting and fast food premises is an area for further research to determine its effectiveness as a public health intervention.

### 4.2. E. coli

*E. coli* is a motile, facultative anaerobic Gram-negative rod-shaped bacteria generally found in the gut of humans and animals, as well as in the environment (soil and water). It has several virulence factors and toxins that contribute to its pathogenicity. It can be flagellated, assisting motility, and some strains produce fimbriae that play a role in attachment to other cells/host tissues. Most *E. coli* are harmless and make up the normal flora inside the intestines [67]. While they are used as a hygiene indicator, i.e., the possibility of faecal contamination, there are specific types that can cause serious food poisoning and, in some cases, can be life threatening.

Pathogenic *E. coli* are classified into specific groups based on how they interact with the host intestinal epithelial cells [68]. Enterotoxigenic *E. coli* (ETEC) strains produce one or both of two distinct enterotoxins responsible for diarrhoea and are the most common cause of travellers’ diarrhoea. Enteroinvasive *E. coli* (EIEC) strains penetrate and multiply within intestinal epithelial cells and produce an enterotoxin responsible for diarrhoea and dysentery symptoms, as in Shigellosis. Enteropathogenic *E. coli* (EPEC) strains limit host immune responses as well as triggering cell destruction, leading to bloody diarrhoea, and they are an important cause of potentially fatal diarrhoea in infants and children in countries without adequate sanitation infrastructure. Enteroaggregative *E. coli* (EAEC) strains adhere in clumps in localised sections of epithelial cells causing diarrhoea, again, in infants and children in countries without adequate sanitation infrastructure, and they are the second most common cause of travellers’ diarrhoea [68].

Enterohaemorrhagic *E. coli* (EHEC) is a subset of Shiga toxin-producing *E.coli* (STEC) strains that can cause haemorrhagic colitis (HE), which in some instances can progress to haemolytic uremic syndrome (HUS), potentially a fatal illness [69]. STECs are characterised by their ability to produce verocytotoxin (Shiga toxins Stx-1 or Stx-2) and their ability to adhere to cell membranes. STECs are often associated with more severe illness and are the only group of *E. coli* that are notifiable in Australian states and territories, with 0157:H7 being the most commonly reported. In Germany, an outbreak of enterohaemorrhagic *E. coli* (EHEC) with the serotype 0104:H4 occurred in May 2011. This is a rare serotype that had only previously been isolated in seven human cases. This strain represents a combination of virulence factors from two different pathogens, which is a concern [70].

As with *S. aureus*, there are emerging issues with multidrug-resistant (MDR) *E. coli,* with the prevalence of plasmid-borne mcr-1 and bla_NDM_ genes undermining the effectiveness of last-line antibiotics for Gram-negative bacteria (carbapenem and colistin) [71]. There is growing concern for the food industry regarding the increasing number of MDR pathogens in foods of animal origin and the risks posed by the severity of the potential MDR zoonotic diseases [71]. An increase in the prevalence of *E. coli* in ready-to-eat foods has the potential to increase exposure to strains that produce Shiga toxins, a highly significant public health risk due to the severity of the disease in individuals. As with both *B. cereus* and *S. aureus*, *E. coli* also has antibiotic-resistant strains that may complicate the treatment for infections, and its increase in prevalence may increase the spread of these strains.

The annual prevalence for *E. coli* showed a negative linear trend over the period July 2009 to June 2022. Its occurrence across the years was statistically significant; however this association depicted a negative change in prevalence over time. This observed decrease in prevalence over the 13 years of the study and the prevalence rate of 0.68% (n = 7319) (95% CI: 0.67% to 0.70%) could have been influenced by the decreased percentage of RTE food categories that had isolations above the satisfactory limit (50%, (n = 20)). This highlights the variation across the food categories and the rarity of *E. coli* being isolated above the satisfactory limit in the sampled RTE foods.

This research finding is contrary to several studies that have found a higher prevalence of *E. coli* in particular food items, especially from developing countries [72], and *E. coli* O157:H7 has been detected in several food products at average percentage prevalence rates of 6.4% in dairy products, 4.7% in meat and beef and 3.11% in vegetables and fruits in the Middle East and North Africa (MENA) region [73]. Research on *E.coli* prevalence in some food items has demonstrated its possible resistance to ampicillin, penicillin and tetracycline [74]. All studies highlight the ongoing importance of *E. coli* and its public health significance; therefore, its isolation in some of the RTE foods sampled in this study means that efforts to prevent and control it in these food types must be maintained and improved. However, the location/region of the studies, the different food types and the likely different food premises types make comparisons difficult, since observed prevalence rates may vary by region and food type and different methods of analysis.

The highest prevalence rates for *E. coli* were observed in cheese/smallgoods/platters (1.62% (n = 308)), RTE seafood (1.27% (n = 1024)) and dairy/eggs (1.12% (n = 89)). There have been various outbreaks associated with the consumption of raw-milk cheeses [75,76,77], and consensus regarding the risk of this type of unpasteurised product continues to be that it is a public health concern. Outbreaks from STEC *E. coli* strains such as in mettwurst in Australia have also highlighted risks in production methods and in precluding legislative interventions around controls for the industry [78,79]. While this study detected a low prevalence rate of *E. coli* in RTE foods from restaurants, cafés and catering and fast food premises types, this foodborne pathogen still needs to be monitored and trended, particularly in potentially high-risk foods.

### 4.3. S. aureus

*Staphylococcus aureus* (*S. aureus*) also forms part of industry and regulatory surveillance and monitoring testing programs in Australia, typically those that are focused on products with human handling steps. *S. aureus* is a Gram-positive, non-sporing spherical bacterium; however, it has the ability to produce staphylococcal enterotoxin (SE), which is the toxin responsible for the majority of food poisoning cases [80]. It is commonly found in the environment, (soil, water and air), and it is also found in the nose and on the skin of humans [81]. *S. aureus* is a facultative anaerobe, which grows at temperatures ranging from 7 to 48 °C, within a pH range of 4.0 to 10.0, and is resistant to freezing. *S. aureus* is unique in its resistance to low water activity, high salt content and osmotic stress, providing it with the capability of thriving in a wide variety of foods, including foods that do not support other food pathogens [81].

The widespread use of antibiotics has led to the emergence of multidrug-resistant bacteria. A number of studies have isolated methicillin-resistant *Staphylococcus aureus* (MRSA) strains from foods such as minced meat, beef luncheon meat, karish cheese [82], raw meat, quick-frozen meat and RTE meats [47]. For the food industry, the additional ability of MRSA to form biofilms on food contact surfaces poses a further issue for control [83]. These adaptations and survival factors are significant for food safety regulators and industry when considering the potential long-term changes in human diets and food-processing environments. Additionally, the impact of extreme weather events such as drought and storms may increase *Staphylococcus*’s prevalence and dispersion in particular locations [23,84]. As with *B. cereus*, *S. aureus* in RTE foods poses a significant public health risk, from its heat-stable toxins, antibiotic (MRSA) resistance and its adaptability and survival in relation to both biofilms and environments. A rise in prevalence may increase the potential sources of toxin exposure and the spread of antibiotic strains.

The annual prevalence trend for *S. aureus* in RTE was similar to that of non-STEC *E. coli* discussed earlier, exhibiting a statistically significant negative linear change in prevalence over time. It showed a decrease in prevalence over the 13 years of the study, with corresponding odds of *S. aureus* being isolated above the satisfactory limit (OR. 0.872; 95% CI, 0.810–0.939, *p* = <0.001). Any detection of *S. aureus* in RTE foods presents a significant food poisoning risk to the public due its toxicological properties, as well as the emergence of antimicrobial resistance in this pathogen [41,44,85,86]. There is a significant focus on the identification of methicillin-resistant *S. aureus* (MRSA) in foods and the associated public health risks [41,44,47,48,82,85,87]. One study of 550 RTE foods from retail markets in China identified that 12.5% (n = 550) of the samples were positive for *S. aureus* with MPN counts of greater than 0.3 [48] and MRSA resistant strains. While the study included the years from 2011 to 2014, the focus was not on changing prevalence trends, but did highlight the public health risk of MRSA in foods [48].

The Australian autumn and spring seasons both showed a statistically significant negative linear relationship per year of the study for *S. aureus* isolated above satisfactory limits. The seasonal variation in *S. aureus* occurrence in foods has already been studied, with one study conducted from 2016 to 2020 analysing 3604 food samples sourced from supermarkets, factories and producers in Italy establishing that winter was associated with a higher risk of detecting *S. aureus* compared to spring, which demonstrated a protective effect [88]. This finding can inform the review of microbiological sampling programs targeting RTE foods, to factor in the observed seasonal variability in the occurrence of *S. aureus* in RTE foods.

When evaluating the negative linear change over time in the prevalence of *S. aureus* in the food categories of sashimi/sushi and pastry/desserts, it is prudent to consider the possible impact of new guidelines introduced by FSANZ in 2016 for foods requiring special care [89], including foods incorporating raw and low-cooked eggs (tiramisu, mousse) and sushi-type products. However, the possible impact of these guidelines, and any other resultant interventions, needs to be evaluated.

### 4.4. Limitations

The high percentage of satisfactory results in comparison to the unsatisfactory ones may have influenced the linear trends observed. Therefore, the addition of the binary logistic regression model for the second part of the investigation was used to provide a more robust analysis as it accounts for other variables and suggests the relationship between the predicators and the three bacteria is unlikely to occur by chance [27].

Potentially impacting the results is the lower sample rates in the initial year (n = 277) and the year of 2019–2020, where COVID-19 pandemic measures in Western Australia resulted in a three-month closure of all the food premises involved in the study. However, while the number of samples was reduced (n = 442), the annual prevalence rate was not significantly different for *E. coli* and *S. aureus*, although for *B. cereus*, it was the second highest year across the study. While the impact of the initial year and the COVID-19 pandemic on the prevalence trends of the study cannot be accurately determined, the overall linear trends suggest that the influence of these years was limited. However, the other impacts of the COVID-19 pandemic need to be taken into consideration when reviewing the results, specifically around the influence of public health campaigns, the interruptions and changes in food supply chains, labour shortages and the additional hygiene measures required of the food industry in the initial phases of the pandemic [90]. Individually or as a mixture, these factors may be drivers in the observed changes in prevalence during the last three years of the study, and this is an area for further investigation.

Selection bias was a consideration around the participating food premises as there were specific businesses that agreed to provide their sample results for the study, and there were smaller sample numbers in the café and takeaway premises types. There was some attrition and additions of food premises over the study period, so a classification of the types of premises was applied to reduce the impact of this bias.

## 5. Conclusions

The analysis of RTE foods from restaurants, cafés and takeaway facilities in Perth WA indicated that there have been changes in prevalence over time between July 2009 to June 2022, for *B. cereus*, *E. coli* and *S. aureus*, and changes in prevalence in different months, seasons, food categories and food premises types. Also of significance were particular years within the study where differences in prevalence for the three bacteria were observed. The data supplied by industry highlight the importance of long-term data for identifying changes in prevalence. The benefit to public health from pooled industry and regulatory data on foodborne pathogens would allow a more comprehensive assessment of potential changes in risk and a foundation for evidence-based decision-making, policy formulation and targeted interventions. In addition, this study, with its overall high rate of satisfactory results, emphasises the importance of food safety programs that include monitoring and surveillance.

As highlighted, there are differences in microbiological standards for RTE foods and a huge range of diversity of RTE foods throughout regions of the world, which directly influences the ability to compare findings across other studies involving prevalence trends. The current microbiological guidelines for RTE foods in Australia do not differentiate between the different types of RTE foods. The varied cuisine and unique food processes in the foods sampled in this study raise the question whether a raw protein product such as sashimi/carpaccio can be compared microbially to raw produce such as mixed salads, or whether either can be compared to a cooked Asian dish or cheeses and smallgoods. This may be an area for further discussion and review, particularly around the current satisfactory limits in Australia and all the different types of RTE foods, especially those served through restaurants, cafés and takeaway-style food premises.

This study identified the potential public health risk posed by the increasing prevalence of *B*. *cereus* in the particular months of January, May, October and November, the Australian spring, the food categories of mixed salads/cold RTE and cheeses/smallgoods/platters and the food premises types of buffets and catering operations. This is a trend worth investigating to understand the possible drivers behind it. Additionally, the potential impact of inadequacies in current and standard laboratory protocols to explicitly differentiate between *B. cereus* and *B. thuringeniesis,* particularly in leafy vegetables and mixed salads, is important to note. Improved differentiation analysis techniques for *B. cereus* and *B. thuringeniesis* may further enhance our understanding of their prevalence increase impacted by growing seasons, biological pesticide use, growing conditions and climate.

## Figures and Tables

**Table 1:** Microbiological analysis methods for determining *E. coli, S. aureus* and *B. cereus* in the study’s ready-to-eat foods samples.

AOAC 991.14-1994	*Coliforms* and *E. coli* counts in food. *E. coli* Petrifilm. The enumeration of *Escherichia coli* in food samples using Neogen Petrifilm. It involves incubation at 35 °C for 48 ± 4 h.
AS 5013.12.1-2004	Food microbiology, Method 12.1: Microbiology of food and animal feeding stuffs—horizontal method for the enumeration of coagulase-positive *Staphylococci* (*Staphylococcus aureus* and other species)—technique using Baird-Parker agar medium
AS 5013.2-2007	Food microbiology, Method 2: Microbiology of food and animal feeding stuffs—horizontal method for the enumeration of *Bacillus cereus*—Colony-count technique at 30 °C (ISO 7932:2004, MOD) [25]

**Table 2:** Applicable limits of *B. cereus*, *E. coli* and *S. aureus* occurrence in RTE foods as defined in the Alberta Health Services Microbial Guidelines for Ready-to-Eat Foods and FSANZ Compendium of Microbiological Criteria for Food, March 2022 edition.

Applicable Standards	Satisfactory Limit	Above Satisfactory Limit
*E. coli* (*non-STEC*)	<10 cfu/g	≥10 cfu/g
Alberta Health Services Microbial Guidelines for Ready-to-Eat Foods. Limit of detection.		
*S. aureus*	<100 cfu/g	≥100 cfu/g
FSANZ Compendium of Microbiological Criteria for Food, March 2022 edition		
*B. cereus*	<100 cfu/g	≥100 cfu/g
FSANZ Compendium of Microbiological Criteria for Food, March 2022 edition		

cfu/g: colony-forming units/g [24,26].

**Table 3:** Food Category Definitions.

Food Category	Definitions
Ready-to-Eat Seafood	Any seafood product that is normally eaten in its raw state with or without the addition of garnish/additional ingredients at service.
	Any seafood product that is consumed after a process of cooking and chilling with or without the addition of sauces/spices or herbs/garnish at service.
Sashimi/Carpaccio/Sushi/Nigiri/Inari Style	Any meat or vegetable product that is thinly sliced and consumed in its raw state at service.
	Small balls or rolls of cold rice with the addition of rice wine vinegar served with raw/cooked seafood, poultry, meat, vegetables, fruit, yaki nori and/or egg.
Cooked Wet Dishes	Seafood, poultry, meats and vegetables cooked for longer periods of time in sauces with herbs/spices.
Cooked Whole Meats	Meat or poultry product cooked whole or in primary cuts such as roasts. Does not include individual steaks.
Soup and Stock	Soup, stock or jus ready to be used.
Purees/Sauces/Dips	Cooked or cold emulsion sauces; dips, pureed or blended.
Western-Style Cooked Dishes	Meals cooked in the Western style that include a number of ingredients on the plate—may include some uncooked items and may comprise meats, poultry, seafood, vegetables, herbs and spices. Primarily English, European and USA-style cuisines.
Asian-Style Cooked Dishes	Meals cooked in the Asian style that include a number of ingredients on the plate—may include some uncooked items and may comprise meats, poultry, seafood, vegetables, herbs and spices. Primarily Japanese, Chinese, Indonesian, Malaysian and Indian cuisines.
Rice, Noodle or Pasta Dishes	Meals based on rice, noodles or pasta with the addition of meats, poultry, seafood, vegetables, herbs and spices, primarily served hot.
Burgers/Sandwiches/Pizza	Bread base with added ingredients/toppings, including meats, poultry, seafood, vegetables, herbs and spices and sauces served hot or cold.
Mixed Salads/Cold RTE Items	Cold dishes that consist primarily of raw produce such as leafy vegetables, herbs, fruits, nuts, spices and cooked chilled vegetables, spices, meats, poultry, seafood, pulses, legumes and additions such as dairy, pasta or rice.
Cheese/Smallgoods/Platters	Smallgoods, cheeses, antipasto items, canape selections.
Pastry/Dessert/Bread	All forms of pastry/desserts/breads.
Fruit/Nuts	Fresh, dried fruit and nuts, compotes.
Vegetables/Herbs	Cooked or raw, includes mushrooms.
Fried Foods	Foods deep-fried in oil such as chips, fish, spring rolls.
Dairy/Eggs	Cream, custards or milk-based items (pasteurised), eggs ready to eat.
Spices	Dried spices used as garnish at service.
Beverage	Juices, smoothies and cocktails.
Other	Not categorised.

**Table 4:** Research questions with described data types, needs and applicable statistical tests to generate results fulfilling each objective.

Research Question	Data Needs	Type of Data	Applicable Tests
Do the target microbes exhibit annual variations in prevalence which exceed applicable satisfactory standards as defined in the Food Standards Australia and New Zealand (FSANZ) Compendium of Microbiological Criteria and the Alberta Health Services Microbial Guidelines for Ready-to-Eat Foods?	Quantitative data on microorganism counts in RTE foods	Continuous variable	Binary logistic regression SPSS + Tukey’s post hoc (R)
	Years	Continuous variable/categorical variable	
	Food category	Categorical covariate	
	Food premises	Categorical covariate	
	Quantitative data on microorganism counts in RTE foods	Continuous variable	Pearson correlation
Do the target microbes exhibit monthly or seasonal prevalence trends in particular months and seasons?	Quantitative data on microorganism counts in RTE foods	Continuous variable	Binary logistic regression SPSS + Tukey’s post hoc (R)
	Years	Continuous variable/categorical variable	
	Food category	Categorical covariate	
	Food premises	Categorical covariate	
	Season	Categorical covariate	
	Month	Categorical covariate	
Do the target microbes exhibit prevalence trends within individual RTE food categories?	Quantitative data on microorganism counts in RTE foods	Continuous variable	Binary logistic regression SPSS + Tukey’s post hoc (R)
	Years	Continuous variable/categorical variable	
	Food category	Categorical covariate	
	Food premises	Categorical covariate	
Do the target microbes exhibit prevalence trends in individual food premises types?	Quantitative data on microorganism counts in RTE foods	Continuous variable	Binary logistic regression SPSS + Tukey’s post hoc (R)
	Years	Continuous variable/categorical variable	
	Food category	Categorical covariate	
	Food premises	Categorical covariate	

**Table 5:** Results from post hoc analysis highlighting the significant variation between groups. (Binary logistic regression model with year as categorical variable).

	Comparison	Coefficient Estimate	SE	t Value	*p* Value
*B. cereus*	2021–22 to 2011–12	0.925	0.383	2.415	0.042
	2015–16 to 2012–13	−1.556	0.468	−3.326	0.028
	2015–16 to 2013–14	−1.148	0.481	−2.385	0.035
	2015–16 to 2014–15	−1.499	0.463	−3.240	0.003
	Vegetables/Herbs—Rice, Noodle or Pasta Dishes	0.281	0.547	0.514	0.009
	Western-Style Cooked Dishes—Spices	−2.311	0.675	−3.422	0.046
*S. aureus*	2021–22 to 2013–14	−15.543	674.352	−0.023	0.038
	Banquets—A la Carte	−0.917	0.248	−3.700	0.002
	Fried Foods—Cheese/Smallgoods/Platters	−16.277	803.793	−0.020	0.016
	Purees/Sauces/Dips—Cooked Whole Meats	−0.270	1.228	−0.220	0.015
	Western-Style Cooked Dishes—Pastry/Dessert/Bread	−0.365	0.579	−0.629	0.026

**Table 6:** Results from binary logistic regression analysis for year and *B. cereus*, *E. coli* and *S. aureus* in each month and Australian season.

Months		B	*p*.	EXP(B) = OR	95% C.I. for OR	
					Lower	Upper
*E. coli*	January	−0.092	0.416	0.912	0.730	1.139
	February	−0.219	0.067	0.803	0.636	1.016
	March	0.148	0.249	1.159	0.902	1.490
	April	−0.005	0.977	0.995	0.727	1.364
	May	−0.124	0.296	0.884	0.701	1.115
	June	−0.164	0.383	0.848	0.586	1.228
	July	−0.649	0.182	0.523	0.202	1.355
	August	−0.154	0.241	0.857	0.663	1.109
	September	−0.192	0.395	0.825	0.530	1.284
	October	0.066	0.751	1.068	0.712	1.602
	November	0.438	0.097	1.549	0.924	2.596
	December	0.019	0.949	1.020	0.560	1.857
*S. aureus*	January	−0.238	0.116	0.788	0.586	1.060
	February	−0.005	0.960	0.995	0.813	1.218
	March	0.025	0.905	1.025	0.680	1.547
	April	−0.265	0.068	0.767	0.577	1.020
	May	−0.365	0.022	0.694	0.508	0.948
	June	0.081	0.564	1.085	0.823	1.430
	July	−0.155	0.234	0.857	0.664	1.105
	August	0.040	0.704	1.040	0.848	1.276
	September	−0.095	0.548	0.910	0.668	1.239
	October	−0.357	0.011	0.700	0.531	0.922
	November	−0.213	0.071	0.808	0.642	1.018
	December	−0.093	0.387	0.912	0.739	1.124
*B. cereus*	January	0.146	0.012	1.157	1.033	1.295
	February	−0.034	0.674	0.967	0.826	1.132
	March	0.017	0.840	1.018	0.859	1.206
	April	0.148	0.067	1.159	0.990	1.358
	May	−0.032	0.626	0.969	0.853	1.100
	June	0.080	0.399	1.084	0.899	1.306
	July	0.084	0.306	1.088	0.926	1.279
	August	−0.065	0.340	0.937	0.820	1.071
	September	0.176	0.016	1.192	1.034	1.375
	October	0.071	0.197	1.074	0.964	1.196
	November	0.179	0.022	1.196	1.026	1.395
	December	−0.028	0.726	0.973	0.834	1.135
Seasons		B	*p*.	EXP(B) = OR	95% C.I. for OR	
					Lower	Upper
*E. coli*	Summer	−0.160	0.040	0.852	0.731	0.993
	Autumn	−0.005	0.949	0.995	0.864	1.147
	Winter	−0.201	0.057	0.818	0.664	1.006
	Spring	0.136	0.244	1.146	0.912	1.440
*S. aureus*	Summer	−0.093	0.149	0.911	0.802	1.034
	Autumn	−0.242	0.008	0.785	0.656	0.940
	Winter	−0.002	0.972	0.998	0.872	1.142
	Spring	−0.236	0.002	0.789	0.680	0.917
*B. cereus*	Summer	0.058	0.148	1.059	0.980	1.145
	Autumn	0.039	0.351	1.040	0.958	1.128
	Winter	0.025	0.577	1.026	0.938	1.122
	Spring	0.129	<0.001	1.138	1.057	1.226

EXP(B) = OR, exponentiation of the B coefficient—Odds Ratio; 95% CI for OR, 95% confidence interval for Odds Ratio.

**Table 7:** The prevalence of *B. cereus* in Australian seasons above satisfactory limits in individual RTE food categories from July 2009 to June 2022.

	Spring			Summer		
Food Category	Prevalence	Lower 95% CI	Upper 95% CI	Prevalence	Lower 95% CI	Upper 95% CI
Asian-Style Cooked Dishes	4.60%	4.04%	5.16%	2.74%	2.44%	3.03%
Beverage	0.00%	0.00%	0.00%	33.33%	−4.39%	71.05%
Burgers/Sandwiches/Pizza	1.69%	1.26%	2.13%	5.10%	4.09%	6.11%
Cheese/Smallgoods/Platters	6.90%	5.12%	8.67%	1.06%	0.85%	1.28%
Cooked Wet Dishes	0.00%	0.00%	0.00%	0.00%	0.00%	0.00%
Cooked Whole Meats	3.49%	2.75%	4.23%	1.79%	1.45%	2.12%
Dairy/Eggs	0.00%	0.00%	0.00%	0.00%	0.00%	0.00%
Fried Foods	0.00%	0.00%	0.00%	1.82%	1.34%	2.30%
Fruit/Nuts	0.00%	0.00%	0.00%	5.26%	2.90%	7.63%
Mixed Salads/Cold RTE	6.85%	5.74%	7.96%	4.86%	4.07%	5.66%
Other	0.00%	0.00%	0.00%	0.00%	0.00%	0.00%
Pastry/Dessert/Bread	4.98%	4.32%	5.63%	4.20%	3.67%	4.74%
Purees/Sauces/Dips	5.66%	4.14%	7.18%	1.41%	1.08%	1.74%
Ready-to-Eat Seafood	5.38%	4.75%	6.01%	3.96%	3.49%	4.42%
Rice, Noodle or Pasta Dishes	2.02%	1.62%	2.42%	2.54%	2.08%	3.00%
Sashimi/Carpaccio/Sushi	1.49%	1.24%	1.75%	4.27%	3.50%	5.05%
Soup and Stock	2.94%	1.95%	3.93%	0.00%	0.00%	0.00%
Spices	33.33%	11.56%	55.11%	0.00%	0.00%	0.00%
Vegetables/Herbs	4.84%	3.63%	6.04%	1.75%	1.30%	2.21%
Western-Style Cooked Dishes	2.50%	2.21%	2.79%	0.98%	0.87%	1.09%
Total	4.12%	3.93%	4.30%	2.85%	2.73%	2.97%
						
	Autumn			Winter		
Food Category	Prevalence	Lower 95% CI	Upper 95% CI	Prevalence	Lower 95% CI	Upper 95% CI
Asian-Style Cooked Dishes	1.01%	0.87%	1.14%	3.56%	3.09%	4.02%
Beverage	0.00%	0.00%	0.00%	0.00%	0.00%	0.00%
Burgers/Sandwiches/Pizza	2.94%	2.24%	3.64%	5.17%	3.84%	6.50%
Cheese/Smallgoods/Platters	2.47%	1.93%	3.01%	7.04%	5.40%	8.68%
Cooked Wet Dishes	6.45%	4.18%	8.72%	0.00%	0.00%	0.00%
Cooked Whole Meats	3.75%	2.93%	4.57%	3.61%	2.84%	4.39%
Dairy/Eggs	0.00%	0.00%	0.00%	23.08%	14.21%	31.95%
Fried Foods	2.56%	1.76%	3.37%	0.00%	0.00%	0.00%
Fruit/Nuts	0.00%	0.00%	0.00%	0.00%	0.00%	0.00%
Mixed Salads/Cold RTE	8.97%	7.57%	10.38%	0.00%	0.00%	0.00%
Other	0.00%	0.00%	0.00%	0.00%	0.00%	0.00%
Pastry/Dessert/Bread	3.38%	2.83%	3.92%	0.00%	0.00%	0.00%
Purees/Sauces/Dips	5.56%	4.07%	7.04%	4.84%	3.63%	6.04%
Ready-to-Eat Seafood	3.08%	2.68%	3.48%	3.29%	2.85%	3.73%
Rice, Noodle or Pasta Dishes	2.27%	1.80%	2.75%	1.23%	0.97%	1.50%
Sashimi/Carpaccio/Sushi	2.56%	2.00%	3.13%	3.85%	2.99%	4.70%
Soup and Stock	0.00%	0.00%	0.00%	0.00%	0.00%	0.00%
Spices	0.00%	0.00%	0.00%	0.00%	0.00%	0.00%
Vegetables/Herbs	1.96%	1.42%	2.50%	2.00%	1.45%	2.55%
Western-Style Cooked Dishes	2.83%	2.48%	3.19%	3.72%	3.25%	4.19%
Total	3.26%	3.10%	3.42%	3.08%	2.93%	3.23%

**Table 8:** Results from binary logistic regression analysis for year and *B. cereus*, *E. coli* and *S. aureus* in each food premises type.

		B	*p*.	EXP(B) = OR	95% C.I. for OR	
					Lower	Upper
*E. coli*	A la Carte	−0.082	0.319	0.921	0.783	1.083
	Buffet	−0.111	0.046	0.895	0.802	0.998
	Banquet/Catering	−0.009	0.937	0.991	0.787	1.248
	Café					
	Fast Food/Takeaway					
	Supply Chain	0.079	0.721	1.082	0.702	1.666
*S. aureus*	A la Carte	−0.114	0.016	0.893	0.814	0.979
	Buffet	−0.138	0.020	0.871	0.775	0.978
	Banquet/Catering	−0.518	0.146	0.596	0.296	1.197
	Café					
	Fast Food/Takeaway					
	Supply Chain					
*B. cereus*	A la Carte	0.057	0.088	1.059	0.991	1.131
	Buffet	0.092	0.002	1.096	1.034	1.163
	Banquet/Catering	0.107	0.042	1.113	1.004	1.233
	Café	−5.978	0.998	0.003	0.000	.
	Fast Food/Takeaway	0.160	0.347	1.173	0.841	1.637
	Supply Chain	−0.133	0.377	0.875	0.651	1.176

EXP(B) = OR, exponentiation of the B coefficient—Odds Ratio; 95% CI for OR, 95% confidence interval for Odds Ratio.

## Data Availability

Restrictions apply to the availability of these data. Data were obtained from third parties. The original contributions presented in the study are included in the article; further inquiries can be directed to the corresponding author.

## Supplementary Materials

The following supporting information can be downloaded at: https://www.mdpi.com/article/10.3390/ijerph21111426/s1, Table S1: Annual prevalence of *B. cereus*, *non-STEC E. coli* and *S. aureus* calculations including confidence intervals; Figure S1: Combined annual prevalence trend of *B. cereus*, *non-STEC E. coli* and *S. aureus* above satisfactory limits in ready-to-eat foods from July 2009 to June 2022; Figure S2: *B. cereus*, *non-STEC E. coli* and *S. aureus* prevalence in Spring over the years of the study; Table S2: Prevalence of *B. cereus*, *non-STEC E. coli* and *S. aureus* for each month from July 2009 to June 2022 including con-fidence intervals; Table S3: Prevalence of *B. cereus*, *non-STEC E. coli* and *S. aureus* for each Australian season from July 2009 to June 2022 including confidence intervals; Table S4: *B. cereus*, *non-STEC E. coli* and *S. aureus* prevalence in each food category including Confidence Intervals.
