# Supplementary material for: Prevalence Trends of Foodborne Pathogens Bacillus cereus, Non-STEC Escherichia coli and Staphylococcus aureus in Ready-to-Eat Foods Sourced from Restaurants, Cafés, Catering and Takeaway Food Premises
Source: Int J Environ Res Public Health. 2024 Oct 27;21(11):1426. doi: 10.3390/ijerph21111426 (PMC11593717; doi:10.3390/ijerph21111426)
Supplement: Supplementary file 1 [file ijerph-21-01426-s001.zip › ijerph-3175230-Supplementary.pdf]

Article

Prevalence Trends of Foodborne Pathogens *Bacillus cereus*, Non-STEC *Escherichia coli* and *Staphylococcus aureus* in Ready-to-Eat Foods Sourced from Restaurants, Cafés, Catering and Takeaway Food Premises

Table S1: Annual prevalence of *B. cereus, non-STEC E. coli* and *S. aureus* calculations including confidence intervals.

					Non STEC *E. coli* Confidence Intervals			
**Financial Year**	**Non STEC *E. coli* Result ≥10 cfu/g**	**Non STEC *E. coli* Result <10 cfu/g**	**Non STEC *E. coli* Total (n) tests**	**Prevalence Non STEC *E. coli***	**SE = σ / √n**	**ME = SE × Z(1.96)**	**Lower bound = Prevalence rate - ME**	**Upper bound = Prevalence rate + ME**
2009 - 2010	1	227	228	0.44%	0.000290	0.000569	0.38%	0.50%
2010 - 2011	4	411	415	0.96%	0.000473	0.000927	0.87%	1.06%
2011 - 2012	2	407	409	0.49%	0.000242	0.000474	0.44%	0.54%
2012 - 2013	10	508	518	1.93%	0.000848	0.001662	1.76%	2.10%
2013 - 2014	1	546	547	0.18%	0.000078	0.000153	0.17%	0.20%
2014 - 2015	3	567	570	0.53%	0.000220	0.000432	0.48%	0.57%
2015 - 2016	8	634	642	1.25%	0.000492	0.000964	1.15%	1.34%
2016 - 2017	8	775	783	1.02%	0.000365	0.000716	0.95%	1.09%
2017 - 2018	5	827	832	0.60%	0.000208	0.000408	0.56%	0.64%
2018 - 2019	2	738	740	0.27%	0.000099	0.000195	0.25%	0.29%
2019 - 2020	1	441	442	0.23%	0.000108	0.000211	0.21%	0.25%
2020 - 2021	1	556	557	0.18%	0.000076	0.000149	0.16%	0.19%
2021 - 2022	4	632	636	0.63%	0.000249	0.000489	0.58%	0.68%
Totals	50	7269	7319	0.68%	0.000080	0.000157	0.67%	0.70%

					S. aureus Confidence Intervals			
**Financial Year**	***S. aureus* Result ≥100 cfu/g**	***S. aureus* Result <100 cfu/g**	***S. aureus* Total (n) tests**	**Prevalence *S. aureus***	**SE = σ / √n**	**ME = SE × Z(1.96)**	**Lower bound = Prevalence rate - ME**	**Upper bound = Prevalence rate + ME**
2009 - 2010	1	221	222	0.45%	0.000302	0.000593	0.39%	0.51%
2010 - 2011	3	409	412	0.73%	0.000359	0.000703	0.66%	0.80%
2011 - 2012	10	399	409	2.44%	0.001209	0.002370	2.21%	2.68%
2012 - 2013	17	493	510	3.33%	0.001476	0.002893	3.04%	3.62%

2013 - 2014	4	543	547	0.73%	0.000313	0.000613	0.67%	0.79%
2014 - 2015	6	567	573	1.05%	0.000437	0.000857	0.96%	1.13%
2015 - 2016	7	631	638	1.10%	0.000434	0.000851	1.01%	1.18%
2016 - 2017	6	777	783	0.77%	0.000274	0.000537	0.71%	0.82%
2017 - 2018	3	829	832	0.36%	0.000125	0.000245	0.34%	0.39%
2018 - 2019	6	728	734	0.82%	0.000302	0.000591	0.76%	0.88%
2019 - 2020	5	437	442	1.13%	0.000538	0.001055	1.03%	1.24%
2020 - 2021	4	554	558	0.72%	0.000303	0.000595	0.66%	0.78%
2021 - 2022	0	616	616	0.00%	0.000000	0.000000	0.00%	0.00%
Totals	72	7204	7276	0.99%	0.000116	0.000227	0.97%	1.01%

					***B. cereus* Confidence Intervals**			
**Financial Year**	***B. cereus* Result ≥100 cfu/g**	***B. cereus* Result <100 cfu/g**	***B. cereus* Total (n) tests**	***B. cereus* Prevalence**	**SE = σ / √n**	**ME = SE × Z(1.96)**	**Lower bound = Prevalence rate - ME**	**Upper bound = Prevalence rate + ME**
2009 - 2010	1	210	211	0.47%	0.000326	0.000639	0.41%	0.54%
2010 - 2011	9	399	408	2.21%	0.001092	0.002140	1.99%	2.42%
2011 - 2012	9	388	397	2.27%	0.001138	0.002230	2.04%	2.49%
2012 - 2013	22	484	506	4.35%	0.001933	0.003788	3.97%	4.73%
2013 - 2014	17	530	547	3.11%	0.001329	0.002604	2.85%	3.37%
2014 - 2015	24	547	571	4.20%	0.001759	0.003448	3.86%	4.55%
2015 - 2016	6	619	625	0.96%	0.000384	0.000753	0.88%	1.04%
2016 - 2017	22	761	783	2.81%	0.001004	0.001968	2.61%	3.01%
2017 - 2018	26	803	829	3.14%	0.001089	0.002135	2.92%	3.35%
2018 - 2019	26	709	735	3.54%	0.001305	0.002557	3.28%	3.79%
2019 - 2020	20	422	442	4.52%	0.002152	0.004218	4.10%	4.95%
2020 - 2021	24	534	558	4.30%	0.001821	0.003569	3.94%	4.66%
2021 - 2022	34	581	615	5.53%	0.002229	0.004369	5.09%	5.97%
Totals	240	6987	7227	3.32%	0.000391	0.000766	3.24%	3.40%

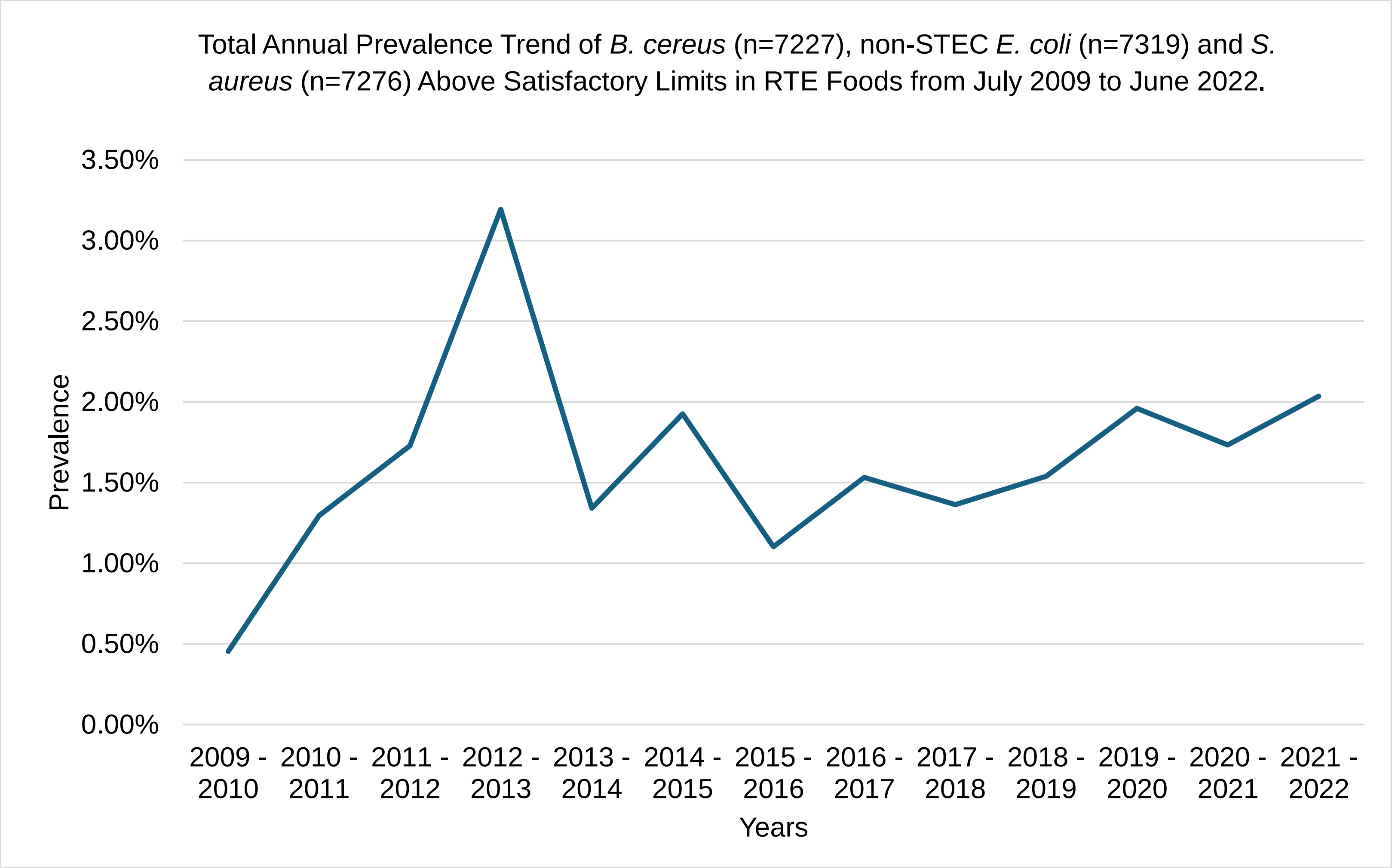

Figure S1: Combined annual prevalence trend of *B. cereus, non-STEC E. coli* and *S. aureus* above satisfactory limits in ready-to-eat foods from July 2009 to June 2022.

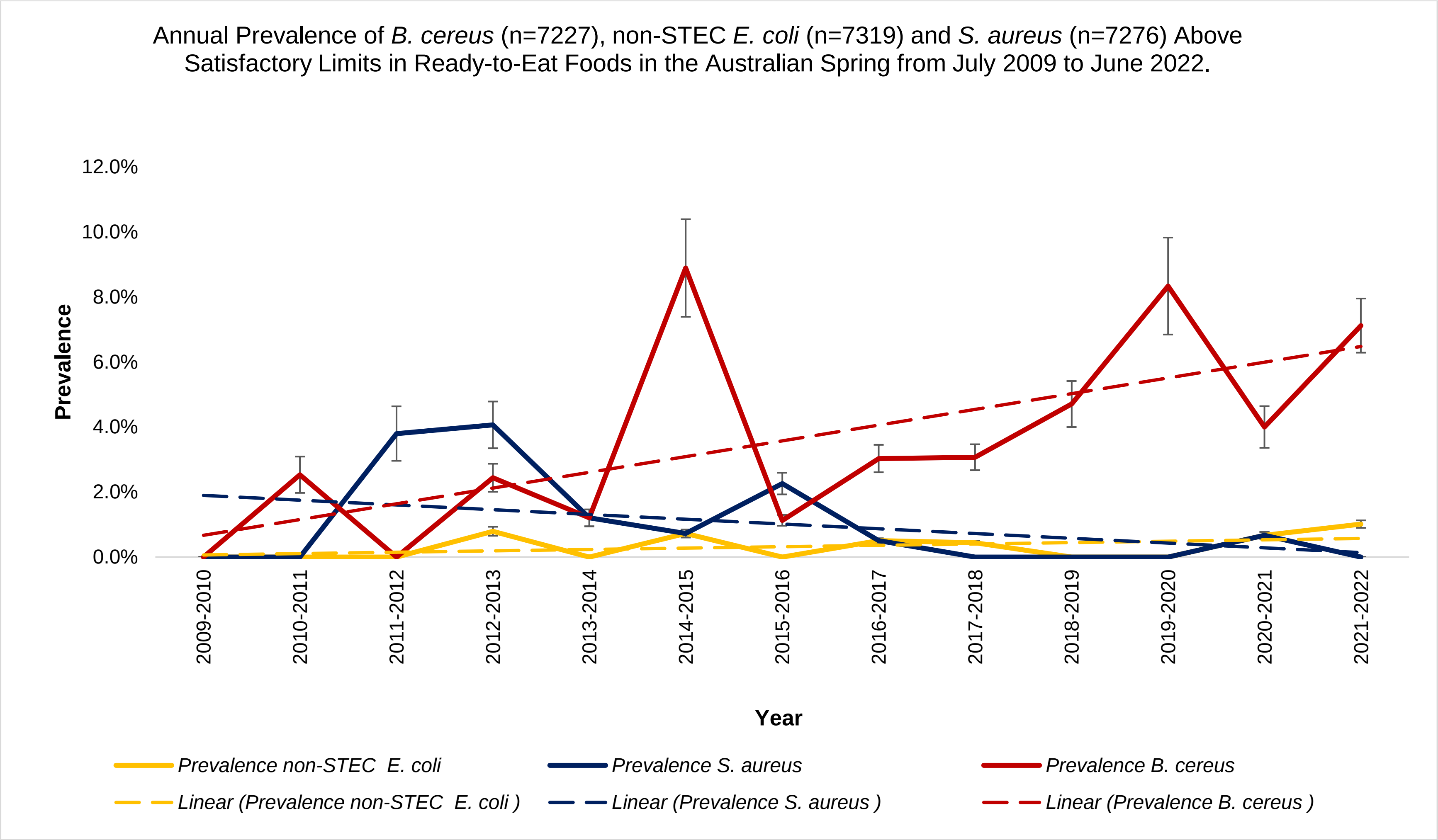

Figure S2: *B. cereus, non-STEC E. coli* and *S. aureus* prevalence in Spring over the years of the study.

Table S2: Prevalence of *B. cereus, non-STEC E. coli* and *S. aureus* for each month from July 2009 to June 2022 including confidence intervals.

					non-STEC *E. coli* Confidence Interval			
	**non-STEC *E. coli* Result >10 cfu/g**	**non-STEC *E. coli* Result <10 cfu/g**	**non-STEC *E. coli* Total (n) tests**	**non-STEC *E. coli* Prevalence**	**SE = σ / √n**	**ME = SE × Z(1.96)**	**Lower bound = Prevalence rate - ME**	**Upper bound = Prevalence rate + ME**
Jan	7	755	762	0.92%	0.000333	0.000652	0.85%	0.98%
Feb	8	603	611	1.31%	0.000530	0.001038	1.21%	1.41%
Mar	6	527	533	1.13%	0.000488	0.000956	1.03%	1.22%
Apr	3	487	490	0.61%	0.000277	0.000542	0.56%	0.67%
May	8	602	610	1.31%	0.000531	0.001041	1.21%	1.42%
Jun	3	515	518	0.58%	0.000254	0.000499	0.53%	0.63%
Jul	1	562	563	0.18%	0.000075	0.000147	0.16%	0.19%
Aug	5	546	551	0.91%	0.000387	0.000758	0.83%	0.98%
Sep	2	659	661	0.30%	0.000118	0.000231	0.28%	0.33%
Oct	2	546	548	0.36%	0.000156	0.000306	0.33%	0.40%
Nov	4	679	683	0.59%	0.000224	0.000439	0.54%	0.63%
Dec	1	788	789	0.13%	0.000045	0.000088	0.12%	0.14%
Totals	50	7269	7319	0.68%	0.000080	0.000157	0.67%	0.70%

					S. aureus Confidence Intervals			
	***S. aureus* Result ≥100 cfu/g**	***S. aureus* Result <100 cfu/g**	***S. aureus* Total (n) tests**	***S. aureus* Prevalence**	**SE = σ / √n**	**ME = SE × Z(1.96)**	**Lower bound = Prevalence rate - ME**	**Upper bound = Prevalence rate + ME**
Jan	5	747	752	0.66%	0.000242	0.000475	0.62%	0.71%
Feb	9	596	605	1.49%	0.000605	0.001185	1.37%	1.61%
Mar	2	534	536	0.37%	0.000161	0.000316	0.34%	0.40%
Apr	5	485	490	1.02%	0.000461	0.000904	0.93%	1.11%

May	7	602	609	1.15%	0.000466	0.000913	1.06%	1.24%
Jun	5	508	513	0.97%	0.000430	0.000843	0.89%	1.06%
Jul	6	557	563	1.07%	0.000449	0.000880	0.98%	1.15%
Aug	9	540	549	1.64%	0.000700	0.001371	1.50%	1.78%
Sep	4	654	658	0.61%	0.000237	0.000464	0.56%	0.65%
Oct	6	529	535	1.12%	0.000485	0.000950	1.03%	1.22%
Nov	6	676	682	0.88%	0.000337	0.000660	0.81%	0.95%
Dec	8	776	784	1.02%	0.000364	0.000714	0.95%	1.09%
Totals	72	7204	7276	0.99%	0.000116	0.000227	0.97%	1.01%

					***B. cereus* Confidence Intervals**			
	***B. cereus* Result ≥100 cfu/g**	***B. cereus* Result <100 cfu/g**	***B. cereus* Total (n) tests**	***B. cereus* Prevalence**	**SE = σ / √n**	**ME = SE × Z(1.96)**	**Lower bound = Prevalence rate - ME**	**Upper bound = Prevalence rate + ME**
Jan	31	720	751	4.13%	0.001506	0.002952	3.83%	4.42%
Feb	15	589	604	2.48%	0.001010	0.001981	2.29%	2.68%
Mar	12	524	536	2.24%	0.000967	0.001895	2.05%	2.43%
Apr	16	473	489	3.27%	0.001480	0.002900	2.98%	3.56%
May	25	574	599	4.17%	0.001705	0.003342	3.84%	4.51%
Jun	11	493	504	2.18%	0.000972	0.001905	1.99%	2.37%
Jul	17	540	557	3.05%	0.001293	0.002535	2.80%	3.31%
Aug	21	510	531	3.95%	0.001716	0.003364	3.62%	4.29%
Sep	27	631	658	4.10%	0.001600	0.003135	3.79%	4.42%
Oct	30	504	534	5.62%	0.002431	0.004765	5.14%	6.09%
Nov	20	659	679	2.95%	0.001130	0.002216	2.72%	3.17%
Dec	15	770	785	1.91%	0.000682	0.001337	1.78%	2.04%
Totals	240	6987	7227	3.32%	0.000391	0.000766	3.24%	3.40%

Table S3: *B. cereus, non-STEC E. coli* and *S. aureus* prevalence in each Australian season with confidence intervals.

	non-STEC E. coli			***S. aureus***			***B. cereus***			**Prevalence**					
**Australian Seasons**	**Result ≥10 cfu/g**	**Result <10 cfu/g**	**Total (n) tests**	**Result ≥100 cfu/g**	**Result <100 cfu/g**	**Total (n) tests**	**Result ≥100 cfu/g**	**Result <100 cfu/g**	**Total (n) tests**	**non-STEC *E. coli***	***S. aureus***	***B. cereus***	**Total above accept limit**	**Total tests**	**%**
Dec-Feb	16	2146	2162	22	2119	2141	61	2079	2140	0.74%	1.03%	2.85%	99	6443	1.54%
Mar-May	17	1616	1633	14	1621	1635	53	1571	1624	1.04%	0.86%	3.26%	84	4892	1.72%
Jun- Aug	9	1623	1632	20	1605	1625	49	1543	1592	0.55%	1.23%	3.08%	78	4849	1.61%
Sep - Nov	8	1884	1892	16	1859	1875	77	1794	1871	0.42%	0.85%	4.12%	101	5638	1.79%
Totals	50	7269	7319	72	7204	7276	240	6987	7227	0.68%	0.99%	3.32%	362	21822	1.66%

non-STEC *E. coli* Confidence Intervals

	**Prevalence**	**Total (n) tests**	**SE = σ / √n**	**ME = SE × Z(1.96)**	**Lower bound = Prevalence rate - ME**	**Upper bound = Prevalence rate + ME**
Summer	0.74%	2162	0.000159	0.000312	0.71%	0.77%
Autumn	1.04%	1633	0.000258	0.000505	0.99%	1.09%
Winter	0.55%	1632	0.000137	0.000268	0.52%	0.58%
Spring	0.42%	1892	0.000097	0.000191	0.40%	0.44%
Totals	0.68%	7319	0.000080	0.000157	0.67%	0.70%

***S. aureus* Confidence Intervals**

	**Prevalence**	**Total (n) tests**	**SE = σ / √n**	**ME = SE × Z(1.96)**	**Lower bound = Prevalence rate - ME**	**Upper bound = Prevalence rate + ME**
Summer	1.03%	2141	0.000222	0.000435	0.98%	1.07%
Autumn	0.86%	1635	0.000212	0.000415	0.81%	0.90%
Winter	1.23%	1625	0.000305	0.000598	1.17%	1.29%
Spring	0.85%	1875	0.000197	0.000386	0.81%	0.89%
Totals	0.99%	7276	0.000116	0.000227	0.97%	1.01%

***B. cereus* Confidence Intervals**

	Prevalence	Total (n) tests	SE = σ / √n	ME = SE × Z(1.96)	Lower bound = Prevalence rate - ME	Upper bound = Prevalence rate + ME
Summer	2.85%	2140	0.000616	0.001208	2.73%	2.97%
Autumn	3.26%	1624	0.000810	0.001587	3.10%	3.42%
Winter	3.08%	1592	0.000771	0.001512	2.93%	3.23%
Spring	4.12%	1871	0.000951	0.001865	3.93%	4.30%
Totals	3.32%	7227	0.000391	0.000766	3.24%	3.40%

Table S4: *B. cereus, non-STEC E. coli* and *S. aureus* prevalence in each food category including Confidence Intervals.

	non-STEC E. coli			***S. aureus***			***B. cereus***			**Prevalence**					
**Food Categories**	**Result ≥10 cfu/g**	**Result <10 cfu/g**	**Total (n) tests**	**Result ≥100 cfu/g**	**Result <100 cfu/g**	**Total (n) tests**	**Result ≥100 cfu/g**	**Result <100 cfu/g**	**Total (n) tests**	**Prevalence non-STEC *E. coli***	**Prevalence *S. aureus***	**Prevalence *B. cereus***	**Total above accept limit**	**Total tests**	**%**
Ready to Eat Seafood	13	1011	1024	8	1001	1009	40	957	997	1.27%	0.79%	4.01%	61	3030	2.01%
Sashimi / Sushi	1	409	410	13	397	410	12	395	407	0.24%	3.17%	2.95%	26	1227	2.12%
Cooked Wet Dishes	0	122	122	1	121	122	2	119	121	0.00%	0.82%	1.65%	3	365	0.82%
Cooked Whole Meats	0	368	368	2	365	367	11	350	361	0.00%	0.54%	3.05%	13	1096	1.19%
Soup and Stock	0	134	134	0	134	134	1	133	134	0.00%	0.00%	0.75%	1	402	0.25%
Purees / Sauces / Dips	0	239	239	1	239	240	10	230	240	0.00%	0.42%	4.17%	11	719	1.53%
Western Style Dishes	3	1083	1086	6	1077	1083	26	1049	1075	0.28%	0.55%	2.42%	35	3244	1.08%
Asian Style Dishes	10	1017	1027	4	1016	1020	31	983	1014	0.97%	0.39%	3.06%	45	3061	1.47%
Rice/ Noodle/ Pasta	2	383	385	2	383	385	8	378	386	0.52%	0.52%	2.07%	12	1156	1.04%
Burgers / Sandwiches	3	291	294	5	286	291	11	272	283	1.02%	1.72%	3.89%	19	868	2.19%
Mixed Salads / Cold RTE	6	575	581	11	565	576	37	538	575	1.03%	1.91%	6.43%	54	1732	3.12%
Cheese / Smallgoods	5	303	308	11	295	306	12	292	304	1.62%	3.59%	3.95%	28	918	3.05%
Pastry / Dessert / Bread	6	748	754	6	748	754	26	724	750	0.80%	0.80%	3.47%	38	2258	1.68%
Fruit / Nuts	0	50	50	0	48	48	1	47	48	0.00%	0.00%	2.08%	1	146	0.68%
Vegetables / Herbs	0	226	226	1	220	221	6	214	220	0.00%	0.45%	2.73%	7	667	1.05%
Fried Foods	0	180	180	0	179	179	2	176	178	0.00%	0.00%	1.12%	2	537	0.37%
Dairy / Eggs	1	88	89	1	88	89	0	87	87	1.12%	1.12%	0.00%	2	265	0.75%
Spices	0	15	15	0	15	15	3	12	15	0.00%	0.00%	20.00%	3	45	6.67%
Other	0	20	20	0	20	20	0	21	21	0.00%	0.00%	0.00%	0	61	0.00%
Beverage	0	7	7	0	7	7	1	10	11	0.00%	0.00%	9.09%	1	25	4.00%
	50	7269	7319	72	7204	7276	240	6987	7227	0.68%	0.99%	3.32%	362	21822	1.66%

non-STEC E. coli Confidence Interval

RTE Food Category	Prevalence	Total (n) tests	SE = σ / √n	ME = SE × Z(1.96)	Upper 95% Confidence Interval	Lower 95% Confidence Interval
Ready to Eat Seafood	1.27%	1024	0.000397	0.000778	1.19%	1.35%
Sashimi / Sushi	0.24%	410	0.000120	0.000236	0.22%	0.27%
Cooked Wet Dishes	0.00%	122	0.000000	0.000000	0.00%	0.00%
Cooked Whole Meats	0.00%	368	0.000000	0.000000	0.00%	0.00%
Soup and Stock	0.00%	134	0.000000	0.000000	0.00%	0.00%
Purees / Sauces / Dips	0.00%	239	0.000000	0.000000	0.00%	0.00%
Western Style Dishes	0.28%	1086	0.000084	0.000164	0.26%	0.29%
Asian Style Dishes	0.97%	1027	0.000304	0.000596	0.91%	1.03%
Rice/ Noodle/ Pasta	0.52%	385	0.000265	0.000519	0.47%	0.57%
Burgers / Sandwiches	1.02%	294	0.000595	0.001166	0.90%	1.14%
Mixed Salads / Cold RTE	1.03%	581	0.000428	0.000840	0.95%	1.12%
Cheese / Smallgoods	1.62%	308	0.000925	0.001813	1.44%	1.80%
Pastry / Dessert / Bread	0.80%	754	0.000290	0.000568	0.74%	0.85%
Fruit / Nuts	0.00%	50	0.000000	0.000000	0.00%	0.00%
Vegetables / Herbs	0.00%	226	0.000000	0.000000	0.00%	0.00%
Fried Foods	0.00%	180	0.000000	0.000000	0.00%	0.00%
Dairy / Eggs	1.12%	89	0.001191	0.002334	0.89%	1.36%
Spices	0.00%	15	0.000000	0.000000	0.00%	0.00%
Other	0.00%	20	0.000000	0.000000	0.00%	0.00%
Beverage	0.00%	7	0.000000	0.000000	0.00%	0.00%
Totals	0.68%	7319	0.000080	0.000157	0.67%	0.70%

***S. aureus* Confidence Intervals**

RTE Food Category	Prevalence	Total (n) tests	SE = σ / √n	ME = SE × Z(1.96)	Upper 95% Confidence Interval	Lower 95% Confidence Interval
Ready to Eat Seafood	0.79%	1009	0.000250	0.000489	0.74%	0.84%
Sashimi / Sushi	3.17%	410	0.001566	0.003069	2.86%	3.48%
Cooked Wet Dishes	0.82%	122	0.000742	0.001455	0.67%	0.97%
Cooked Whole Meats	0.54%	367	0.000284	0.000558	0.49%	0.60%
Soup and Stock	0.00%	134	0.000000	0.000000	0.00%	0.00%
Purees / Sauces / Dips	0.42%	240	0.000269	0.000527	0.36%	0.47%
Western Style Dishes	0.55%	1083	0.000168	0.000330	0.52%	0.59%
Asian Style Dishes	0.39%	1020	0.000123	0.000241	0.37%	0.42%
Rice/ Noodle/ Pasta	0.52%	385	0.000265	0.000519	0.47%	0.57%
Burgers / Sandwiches	1.72%	291	0.001007	0.001974	1.52%	1.92%
Mixed Salads / Cold RTE	1.91%	576	0.000796	0.001560	1.75%	2.07%
Cheese / Smallgoods	3.59%	306	0.002055	0.004028	3.19%	4.00%
Pastry / Dessert / Bread	0.80%	754	0.000290	0.000568	0.74%	0.85%
Fruit / Nuts	0.00%	48	0.000000	0.000000	0.00%	0.00%
Vegetables / Herbs	0.45%	221	0.000304	0.000597	0.39%	0.51%
Fried Foods	0.00%	179	0.000000	0.000000	0.00%	0.00%
Dairy / Eggs	1.12%	89	0.001191	0.002334	0.89%	1.36%
Spices	0.00%	15	0.000000	0.000000	0.00%	0.00%
Other	0.00%	20	0.000000	0.000000	0.00%	0.00%
Beverage	0.00%	7	0.000000	0.000000	0.00%	0.00%
Totals	0.99%	7276	0.000116	0.000227	0.97%	1.01%

B. cereus Confidence Intervals

RTE Food Category	Prevalence	Total (n) tests	SE = σ / √n	ME = SE × Z(1.96)	Upper 95% Confidence Interval	Lower 95% Confidence Interval
Ready to Eat Seafood	4.01%	997	0.001271	0.002490	3.76%	4.26%
Sashimi / Sushi	2.95%	407	0.001461	0.002864	2.66%	3.23%
Cooked Wet Dishes	1.65%	121	0.001503	0.002945	1.36%	1.95%
Cooked Whole Meats	3.05%	361	0.001604	0.003143	2.73%	3.36%
Soup and Stock	0.75%	134	0.000645	0.001264	0.62%	0.87%
Purees / Sauces / Dips	4.17%	240	0.002690	0.005272	3.64%	4.69%
Western Style Dishes	2.42%	1075	0.000738	0.001446	2.27%	2.56%
Asian Style Dishes	3.06%	1014	0.000960	0.001882	2.87%	3.25%
Rice/ Noodle/ Pasta	2.07%	386	0.001055	0.002068	1.87%	2.28%
Burgers / Sandwiches	3.89%	283	0.002311	0.004529	3.43%	4.34%
Mixed Salads / Cold RTE	6.43%	575	0.002683	0.005260	5.91%	6.96%
Cheese / Smallgoods	3.95%	304	0.002264	0.004437	3.50%	4.39%
Pastry / Dessert / Bread	3.47%	750	0.001266	0.002481	3.22%	3.71%
Fruit / Nuts	2.08%	48	0.003007	0.005894	1.49%	2.67%
Vegetables / Herbs	2.73%	220	0.001839	0.003604	2.37%	3.09%
Fried Foods	1.12%	178	0.000842	0.001651	0.96%	1.29%
Dairy / Eggs	0.00%	87	0.000000	0.000000	0.00%	0.00%
Spices	20.00%	15	0.051640	0.101214	9.88%	30.12%
Other	0.00%	21	0.000000	0.000000	0.00%	0.00%
Beverage	9.09%	11	0.027410	0.053724	3.72%	14.46%
Totals	3.32%	7227	0.000391	0.000766	3.24%	3.40%
